# MtDNA Haplogroup A10 Lineages in Bronze Age Samples Suggest That Ancient Autochthonous Human Groups Contributed to the Specificity of the Indigenous West Siberian Population

**DOI:** 10.1371/journal.pone.0127182

**Published:** 2015-05-07

**Authors:** Aleksandr S. Pilipenko, Rostislav O. Trapezov, Anton A. Zhuravlev, Vyacheslav I. Molodin, Aida G. Romaschenko

**Affiliations:** 1 Institute of Cytology and Genetics, Siberian Branch of Russian Academy of Sciences, Novosibirsk, Russia; 2 Institute of Archaeology and Ethnography, Siberian Branch of Russian Academy of Sciences, Novosibirsk, Russia; 3 Novosibirsk State University, Novosibirsk, Russia; Universitat Pompeu Fabra, SPAIN

## Abstract

**Background:**

The craniometric specificity of the indigenous West Siberian human populations cannot be completely explained by the genetic interactions of the western and eastern Eurasian groups recorded in the archaeology of the area from the beginning of the 2^nd^ millennium BC. Anthropologists have proposed another probable explanation: contribution to the genetic structure of West Siberian indigenous populations by ancient human groups, which separated from western and eastern Eurasian populations before the final formation of their phenotypic and genetic features and evolved independently in the region over a long period of time. This hypothesis remains untested. From the genetic point of view, it could be confirmed by the presence in the gene pool of indigenous populations of autochthonous components that evolved in the region over long time periods. The detection of such components, particularly in the mtDNA gene pool, is crucial for further clarification of early regional genetic history.

**Results and Conclusion:**

We present the results of analysis of mtDNA samples (n = 10) belonging to the A10 haplogroup, from Bronze Age populations of West Siberian forest-steppe (V—I millennium BC), that were identified in a screening study of a large diachronic sample (n = 96). A10 lineages, which are very rare in modern Eurasian populations, were found in all the Bronze Age groups under study. Data on the A10 lineages’ phylogeny and phylogeography in ancient West Siberian and modern Eurasian populations suggest that A10 haplogroup underwent a long-term evolution in West Siberia or arose there autochthonously; thus, the presence of A10 lineages indicates the possible contribution of early autochthonous human groups to the genetic specificity of modern populations, in addition to contributions of later interactions of western and eastern Eurasian populations.

## Introduction

The West Siberian Plain covers a vast area within northern Eurasia: from the Ural Mountains in the west to the Yenisei River in the east. The region, along with Central Asia, has traditionally been considered to form part of a contact area between western and eastern Eurasian human populations [1, 2, 3, 4]. West Siberia is the place of origin of numerous human ethnic groups, including the Uralic-speaking Ugric and Samoyed populations inhabiting the northern part of the West Siberian Plain, and Turkic populations (Siberian Tatars) inhabiting the southern part. The specificity of modern indigenous populations of the region is evidenced by their linguistic affiliations and their physical anthropological traits. Most of the modern indigenous human groups of West Siberia reveal traits that are specific of a so-called Ural anthropological type [5] (or West Siberian anthropological type [1]), such as a peculiar combination of features characteristic of western and eastern Eurasian groups. Archaeological, anthropological and genetic data (mixed pattern in the gene pool of modern populations) suggest, without doubt, that long interaction and reciprocal genetic influence between groups with western Eurasian and eastern Eurasian origin were crucial mechanisms in the evolution of West Siberian indigenous human groups[1, 2, 3, 4]. However, the role of another hypothesized factor remains unclear: the contribution to their genetic structure of ancient human groups that early separated from other western Eurasian and eastern Eurasian populations, and evolved independently in the region over a long period of time [6].

From the paleoanthropological point of view, the ancient substrate could be associated with human groups belonging to the so-called ‘northern Eurasian anthropological formation’ that arose in the region by the Neolithic period [2, 6]. But specific potentially autochthonous genetic components, which may confirm this hypothesis, have not been identified to date in the mtDNA gene pool of modern West Siberian populations and thus, detection of such components is crucial for further clarification of the early regional genetic history.

Paleogenetic approaches allow the direct study of the gene pool of regional ancestral populations, and facilitate the search for potentially indigenous genetic features. Ancient DNA studies also allow us to investigate ancient genetic components, the role of which in the gene pool was subsequently strongly modified by genetic drift [7, 8]. Ancient DNA approaches have already been applied to the reconstruction of genetic history of Siberian populations, in research on the genetic characteristics of earliest anatomically modern humans from East Siberia [9], the recent admixture of populations in the Southern Siberia [10] and other topics.

**Fig 1:**
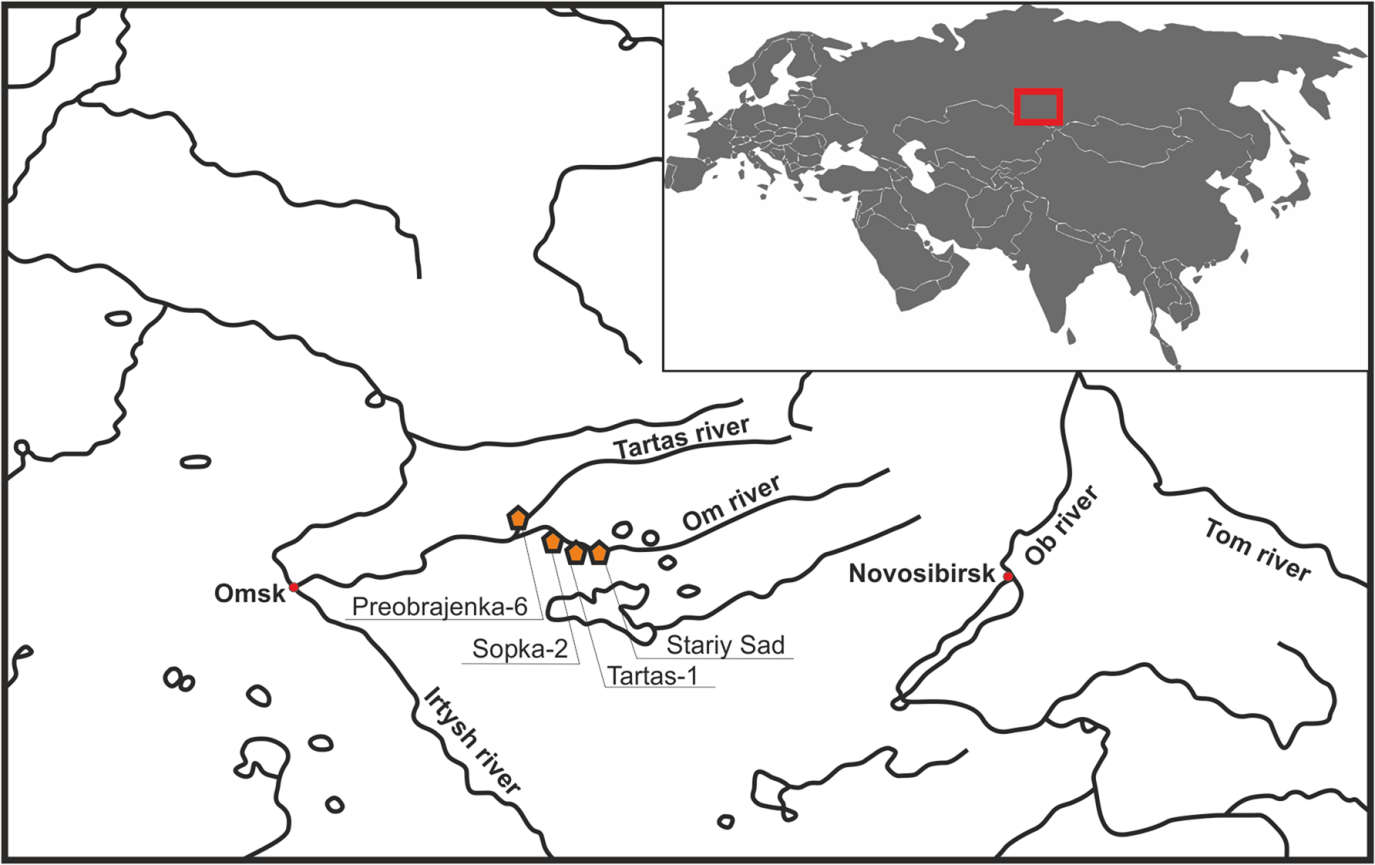
Location of the archaeological sites from which samples for this study were obtained.

In this paper, we present the results of analysis of mtDNA samples (n = 10) belonging to the A10 haplogroup, from West Siberian forest-steppe zone human populations dating from the Early Metal Age to the Late Bronze Age (the end of 5^th^ to the beginning of 1^st^ millennium BC) (see Tables 1 and 2 and Materials and Methods for details). These specimens were identified in the study of a large diachronic sample (n = 96) from the Baraba region, located between the Ob and Irtysh Rivers (Fig 1). The ancient Baraba forest-steppe populations showed craniometric features similar to those of the Ural (West Siberian) anthropological type since the Neolithic period [2, 11]. Data on the distribution and variability of A10 mtDNA lineages in ancient West Siberian and modern Eurasian human populations are considered in light of the possible contribution of ancient autochthonous genetic substrates (‘proto-Uralic’ or ‘proto-West-Siberian’) to the early evolutionary stages of modern indigenous West Siberian human populations (including Ugric and Samoyed), and to the formation of their genetic specificity.

**Table 1:** The Bronze Age cultural groups from West Siberian forest-steppe region analyzed in this study (see also S2 File).

Cultural Group	Age (dating method)	Archaeological Period	Area in Eurasia
Ust-Tartas Culture	The end of V—the middle of IV millennium BC (C^14^)	The Early Metal Age (Eneolithic)	The forest-steppe zone between Ob and Irtysh rivers (Baraba forest-steppe region)
Odinovo Culture	III millennium BC (C^14^)	The Early and beginning of the Middle Bronze Age	Forest-steppe zone between Ishim and Irtysh rivers and Western Part of the Baraba forest-steppe
Krotovo Culture	III millennium BC (C^14^)	Beginning of the Middle Bronze Age	The forest-steppe zone between Ob and Irtysh rivers (Baraba forest-steppe region) and adjacent territories
Late Krotovo Culture	20–18 centuries BC (C^14^)	The Middle Bronze Age	Western Part of the Baraba forest-steppe
Andronovo (Fedorovo) Culture	20–15 centuries BC (C^14^)	The Middle Bronze Age	Forest-steppe and steep zones of Kazakstan, Eastern Urals and West Siberia
Pakhomovo Culture	14–8 centuries BC (Archaeological dating)	The Late Bronze Age	Forest-steppe zone from Eastern Urals to western part of Baraba region

**Table 2:** Description of paleoanthropological materials analyzed in this study (see also S2 File).

Sample Name	Burial Ground	Burial (Skeleton) number	Cultural group	Skeletal material studied	Date
Ut5	Sopka-2/3	655 (D4)	Ust-Tartas	humerus, tibia	1^st^ half—the middle of 4th millennium BC
Ut38	Sopka-2/3	655 (B)	Ust-Tartas	tibias	1^st^ half—the middle of 4th millennium BC
Krz1	Sopka-2/4a	210 (A)	Odinovo	teeth	23–22 centuries BC
Od7	Preobrajenka-6	48	Odinovo	tibias	23–20 centuries BC
Od11	Preobrajenka-6	10	Odinovo	tibias	23–20 centuries BC
Krb10	Sopka-2/4b	177	Krotovo	tibias	28–24 centuries BC
TK10	Tartas-1	76	Late Krotovo	humerus	19–18 centuries BC
TA17	Tartas-1	189	Andronovo (Fedorovo)	tibia, teeth	18–14 centuries BC
Sts6	Stary Sad	6–1 (1)	Pakhomovo	tibias	14–8 centuries BC
Sts11	Stary Sad	49–1	Pakhomovo	tibias	14–8 centuries BC

## Materials and Methods

### The ancient human groups of the Baraba forest-steppe region under study

The paleoanthropological materials under study were excavated from several West Siberian Bronze Age archaeological sites located in the north of the Baraba forest-steppe between the Ob and Irtysh rivers, and near the border of the forest-steppe and taiga zones. The region is archaeologically well studied: intensive excavations have been carried out over the past 40 years. As a result, a large set of data is available concerning the ancient ethno-cultural groups that occupied this territory from the Neolithic period (VII–VI millennium BC or even earlier) to the Late Middle Ages. The most representative materials, including collections of paleoanthropological remains, were obtained for all Baraba populations from the Bronze Age [12, 13]. The large burial grounds are associated with the main regional Bronze Age ethno-cultural groups located in the north of Baraba forest-steppe (Fig 1). We studied the populations of the Early Metal and Bronze Age; their chronology is presented in Table 1. In addition to archaeological descriptions, detailed descriptions of characteristics of these populations from physical anthropology were obtained [2]. For this research it is particularly important that early Baraba populations from the Neolithic and Early Bronze Age periods before the arrival of the Andronovo groups in the first half of II millennium BC (namely the Ust-Tartas, Odinovo and Krotovo groups), showing cranial features corresponding to the Uralic (or West Siberian) anthropological type, and belonging to the Northern Eurasian anthropological formation, are used. Using these anthropological materials, we carried out a screening study of the ancient mtDNA gene pool structure and its dynamics in the West Siberian forest-steppe zone, including samples from about 100 individuals to date (some of the preliminary results were published [12, 14]). Only 10 samples that turned out to belong to haplogroup A10 after the initial screening were further used in this study.

### Paleoanthropological samples

All specimens analyzed in this study were obtained from Repository of paleoanthropological collections of Institute of Archaeology and Ethnography SB RAS (Novosibirsk, Russia). All specimens are available for possible future studies under its whole description, including the name of archaeological site (burial ground name), number of burial (and skeleton number for double or collective burials). Whole descriptions of the specimens under study are given in Table 2. All specimens are available for investigation under informal permission of the author of the archaeological excavations. The author of excavation of all specimens under this study is Vyacheslav I. Molodin—participant of our study and one of the co-authors of this manuscript. Thus, no additional permits were required for the described study.

Climatic conditions in the Baraba region are favorable for the preservation of skeletal material and the DNA therein. All skeletal remains investigated were macroscopically well-preserved. For each individual, at least two samples from the same or different post-cranial bones, or several teeth, were collected and studied separately (Table 2). Most samples were obtained from the skeletons, which have been excavated years ago and stored in the Repository. Additionally, samples from individual TA17 (Andronovo period, See Table 2) were taken in the field during excavations by one of the authors (P.A.S.), in which sterility criteria were followed.

Ancient DNA (aDNA) experiments were carried out in the Laboratory of molecular paleogenetics in the Institute of Cytology and Genetics SB RAS (Novosibirsk, Russia).

### DNA extraction

DNA was extracted as previously described [15, 16]. In brief, surfaces of bone and tooth samples were cleaned mechanically, and then treated with a 7% bleach solution and irradiated by UV light (for at least 1 hour on each side of each sample). Powder was drilled from the internal compact tissue of bones, or from the pulp cavities of teeth. DNA was extracted from bone powder by means of incubation in a 5M guanidinium thiocyanate (GuSCN) buffer (pH 8.0) for 48 h at 65°C, followed by phenol/chlorophorm extraction and precipitation with isopropanol. Tooth powder was decalcified by treatment with 0.5 M EDTA solution, then incubated with proteinase K, after which the extraction and precipitation procedures described above were followed. Two to four extractions were performed for each individual under study.

### Mitochondrial DNA analysis

Four overlapping fragments of mtDNA hyper-variable region (HVR) I and a coding-region fragment containing the A663G polymorphism which is characteristic for haplogroup A were amplified and sequenced using primers from [7, 17] (S1 Table). The PCR-fragments were checked on a 4% polyacrylamide gel. A set of PCR products was cloned with the pGEM-T Easy Vector System Kit (Promega, USA) and 5–8 colonies for each cloned fragment were amplified with M13 universal primers and sequenced. The other PCR-fragments were sequenced directly. Sequencing reactions were performed with an ABI Prism BigDye Terminator Cycle Sequencing Ready Reaction Kit (Applied Biosystems, USA) and were analyzed on an ABI Prism 3130XL Genetic Analyzer (Applied Biosystems, USA) at the SB RAS Genomics Core Facility (Novosibirsk, Russia).

Sequences were aligned with the revised Cambridge Reference Sequence of human mtDNA (rCRS) [18] by using BioEdit software version 7.0.5. Phylogenetic interpretation of sequences was performed according to the current classification of mtDNA variability (mtDNA tree Build 15, http://www.phylotree.org) [19]. A phylogenetic network among ancient and modern A10 haplotypes was constructed by using the Network program v. 4.6.1.1. (www.fluxus-enginering.com). Phylogeographic analysis was conducted by using a database of mtDNA variability including more than 30,000 samples from modern Eurasian populations collected from published sources (see S1 File).

### Sex determination and autosomal STR analysis

Nine autosomal STRs and the sex-determining marker amelogenin were simultaneously co-amplified by using the AmpFlSTR Profiler Plus Kit (Applied Biosystems, USA) according to the manufacturer`s instructions. Results were analyzed on an ABI Prism 3130XL Genetic Analyzer (Applied Biosystems, USA) at the SB RAS Genomics Core Facility (Novosibirsk, Russia).

### Precautions against contamination

Work with the ancient material was carried out in specially equipped, isolated clean rooms using special clothes. All work surfaces and instruments were routinely cleaned with a 5% solution of bleach and irradiated by UV light. Blank controls were run in parallel with samples in all extraction and amplification procedures, to identify possible contamination. MtDNA HVRI sequences were determined for all staff working with the ancient DNA, as well as for anthropologists and archaeologists who may have had contact with the study material.

## Results

Sequences of the mtDNA HVR I fragment (15997–16409) were obtained for all the individuals under study. Differences between each ancient mtDNA sample and the rCRS are shown in Table 3 (see also S2 Table). The motif 16223T, 16227C, 16290T, 16311C, and 16319A is common to all the samples. The motif 16223T, 16290T, and 16319A suggests that the samples belong to East Eurasian haplogroup A. This classification is also supported by the presence of transition A663G in the coding region of the mtDNA, which marks all lineages of this haplogroup. The mtDNA HVRI haplotype 16223T, 16227C, 16290T, 16311C, 16319A occurs only once in a global human mtDNA phylogenetic tree, in subcluster A10, and it is the root lineage of this mtDNA haplogroup [19] (the cluster was first included in PhyloTree Build 7 (10 Nov 2009); thereafter it was also reported in [20] as A8b). Therefore, all mtDNA samples studied are unambiguously attributed to the A10 haplogroup.

**Table 3:** Structure of mtDNA haplogroup A10 lineages from Baraba Bronze Age individuals analyzed in this study.

Sample name	HVR I (15996–16410) haplotype	Nucleotide at position 663	Subcluster
Ut5	16148T, 16223T, 16227C, 16290T, 16311C, 16319A	G	A10*
Ut38	16148T, 16223T, 16227C, 16290T, 16311C, 16319A	G	A10*
Krz1	16223T, 16227C, 16230G, 16290T, 16311C, 16319A	G	A10a
Od7	16223T, 16227C, 16290T, 16311C, 16319A	G	A10*
Od11	16223T, 16227C, 16290T, 16311C, 16319A	G	A10*
Krb10	16223T, 16227C, 16290T, 16311C, 16319A	G	A10*
TK10	16223T, 16227C, 16290T, 16311C, 16319A	G	A10*
TA17	16223T, 16227C, 16278T, 16290T, 16311C, 16319A	G	A10*
Sts6	16093C, 16223T, 16227C, 16290T, 16311C, 16319A	G	A10*
Sts11	16148T, 16223T, 16227C, 16290T, 16311C, 16319A	G	A10*

Phylogenetic analyses of A10 mtDNA lineages published in the scientific literature to date (and discussed in detail below) reveal that most of them can be grouped into a subcluster within haplogroup A10, with common haplotype 16223T, 16227C, 16230G, 16290T, 16311C, 16319A. We provisionally named this subcluster A10a, in accordance with the rules of nomenclature of mtDNA phylogenetic clusters. In the remainder of this paper we will refer to A10 mtDNA lineages without the A16230G transition as A10*. The precise phylogenetic substructure of the A10 haplogroup can be fully elucidated only by complete mtDNA genome sequencing. The overall frequency of A10 in the West Siberian Bronze Age population that we studied was at least ~8.5% (see details in S1 File).

**Fig 2:**
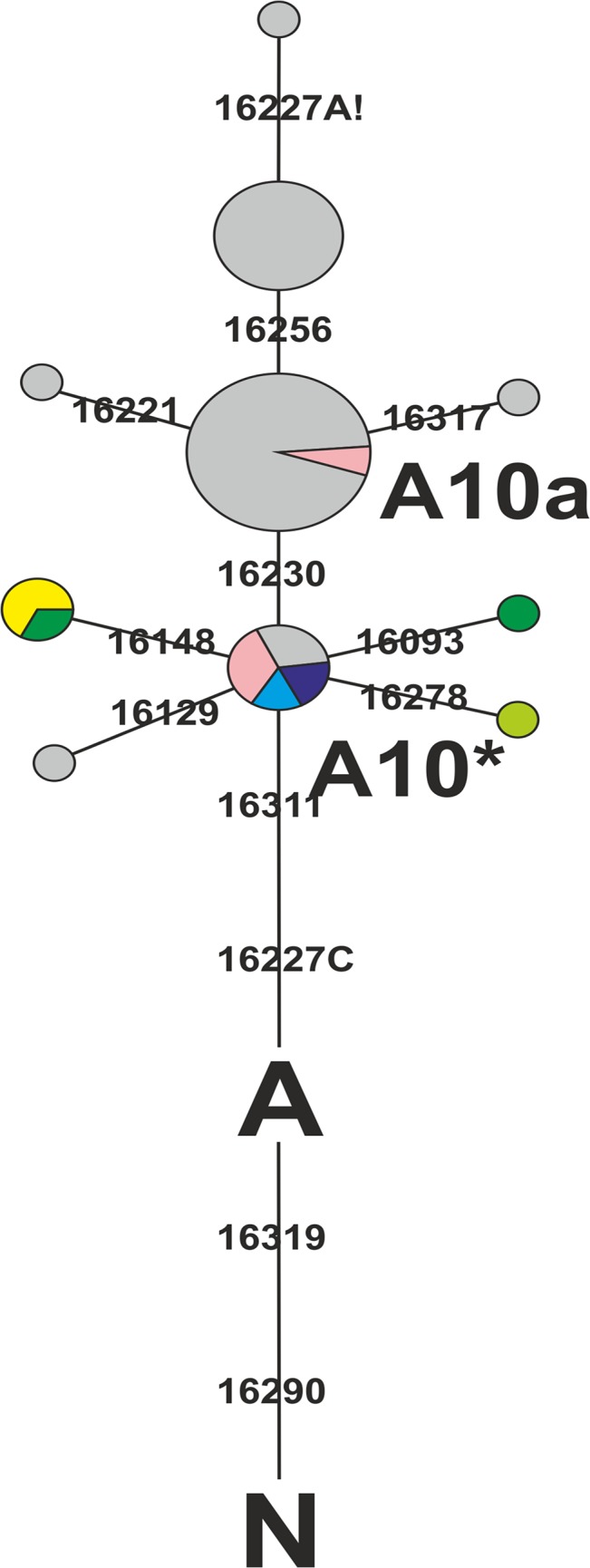
Phylogenetic tree of mtDNA haplogroup A10 lineages from modern and ancient populations of Eurasia. Ascription to the populations showed by colors: grey—modern populations of Eurasia (see S3 Table for details); yellow—Ust-Tartas culture; pink—Odinovo culture; blue—Krotovo culture; dark blue—Late Krotovo culture; green—Andronovo (Fedorovo) culture; dark green—Pakhomovo culture.

A total of five different HVR I haplotypes were revealed among the ten ancient mtDNA samples that we studied (Table 3, Fig 2), with four belonging to the A10* cluster and one a root haplotype of the A10a subcluster. The presence of root and/or diverged lineages of the A10* subcluster was detected in all Bronze Age populations from the Baraba region under study (Table 3). The root variant of the A10 (and A10*) haplogroup, with haplotype structure 16223T, 16227C, 16290T, 16311C, 16319A, was the most commonly represented lineage among the samples. It was revealed in four specimens belonging to several periods of the Bronze Age, from the Early Bronze Age (the Odinovo culture, III millennium BC) to the end of the Middle Bronze Age (the Late Krotovo culture, the middle of II millennium BC). The simultaneous presence of A10* and A10a was detected in the gene pool of an Early Bronze Age population (the Odinovo culture, III millennium BC), which is the earliest (and only) case of the presence of the A10a lineage in the ancient West Siberian populations that we have studied.

Therefore, the presence of mtDNA haplogroup A10 lineages in all the main Bronze Age populations of the Baraba forest-steppe region was revealed. Several directions of haplogroup A10 diversification from the root haplotype were found. Lineages with the 16230 transition (A10a subgroup in our terms) were only one component of A10 variability in West Siberian Bronze Age populations, and were relatively rare.

**Fig 3:**
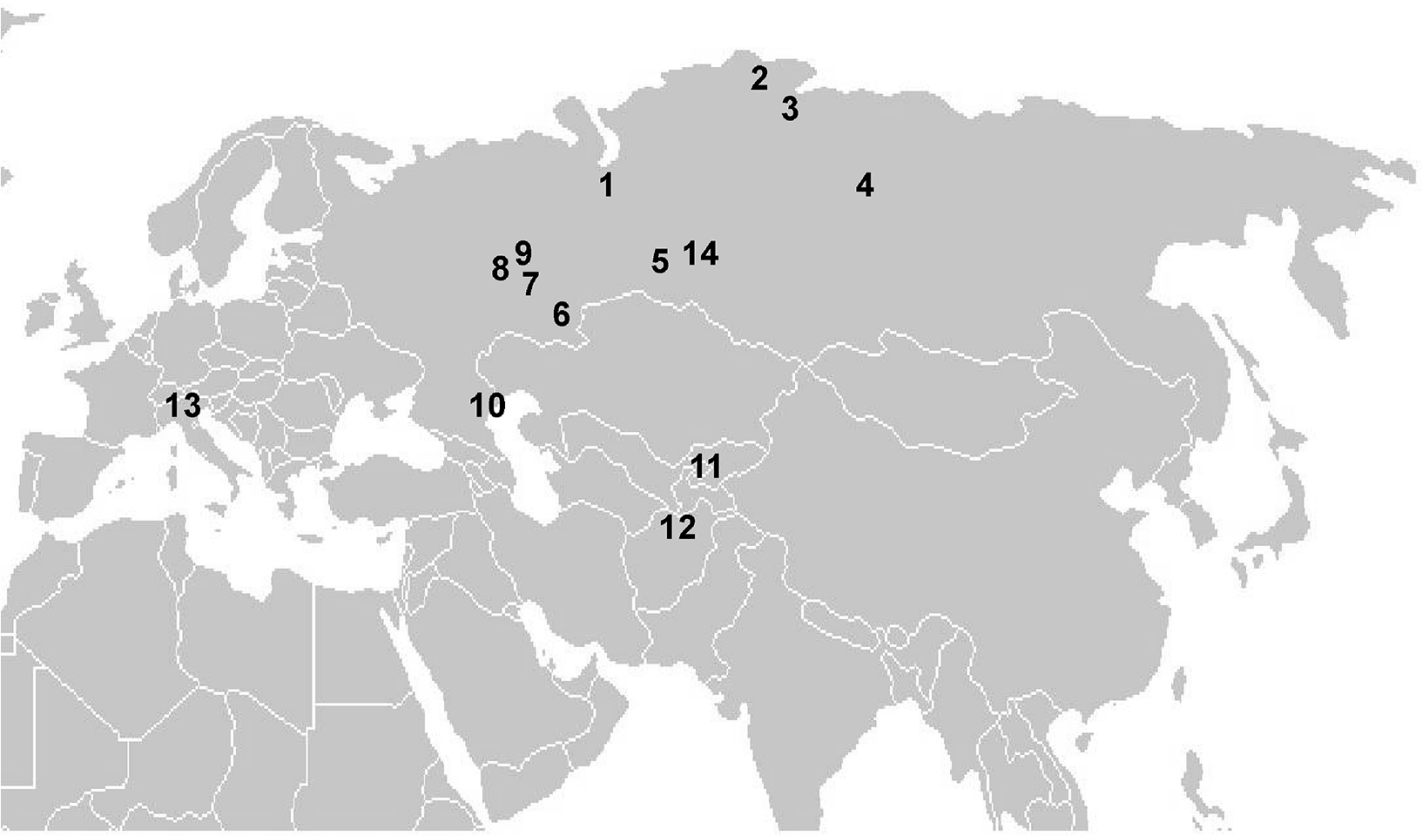
Geographic localization of modern Eurasian human populations with mtDNA haplogroup A10 lineages in gene pools. 1—Mansi [21]; 2—Nganasans [22]; 3—Dolgans [22]; 4—Evenks [22]; 5—Siberian Tatars [23]; 6—Bashkirs [24]; 7—Tatars [18, 24]; 8—Chuvash [24]; 9—Mari [24]; 10—Nogays [25]; 11—Tadjiks [22]; 12—Afghanistan Hazara and Uzbeks [20]; 13—population of Italian Alps [19]; 14—ancient Baraba forest-steppe populations.

The modern indigenous populations of Eurasia differ from ancient West Siberian human groups in the distribution and phylogenetic structure of A10 lineages in their gene pools. Generally, the mtDNA haplogroup A10 is a rare component of the gene pool of current Eurasian human populations (see S1 File). We found 33 individuals with A10 lineages in the data set used for phylogeographic analysis (see S3 Table). Most individuals with A10 mtDNA lineages were found in populations from central Eurasia: from the Volga-Ural region to East Siberia, and from the north of West Siberia to the south of Central Asia (Fig 3); thus the current area of haplogroup A10 includes all of West Siberia and adjacent territories. The Baraba forest-steppe region is central in this area. A10 lineages are sporadically and unevenly distributed in contemporary indigenous populations within the overall area. In many populations, A10 lineages are completely absent, and most of the A10 carriers were from the northern part of the area indicated above: the north of the steppe, forest-steppe and taiga zones. Frequencies of A10 haplogroup in the gene pool of most contemporary populations do not exceed 1–3% (S3 Table). The overall frequency of A10 lineages in the regional modern indigenous populations is even significantly lower if we take into account populations in which A10 haplogroup was not detected (see S1 File).

Modern Eurasian populations differ strongly in the phylogenetic structure of their haplogroup A10 lineages from West Siberian Bronze Age human groups. We identified only seven different A10 haplotypes in contemporary Eurasians. All lineages found within the main current area of the A10 haplogroup (five lineages of a total of seven) share motifs 16223T, 16227C, 16230G, 16290T, 16311C, 16319A, and therefore belong to the subcluster A10a (Fig 3). The rare lineages belonging to A10* were found in populations from the Italian Alps [21] and from Afghanistan [22], outside the main area of the haplogroup. The root A10 haplotype, which was the most common one in the Bronze Age populations of West Siberia, is present in our database only in modern indigenous populations of Afghanistan [22].

Furthermore, some phylogenetic differences in A10 lineages are common in the western and eastern parts of the whole area. In the north-eastern part of the area (including eastern and northern parts of West Siberia and East Siberia), haplogroup A10 is represented almost solely by the A10a subhaplotype 16223T, 16227C, 16230G, 16256T, 16290T, 16311C, 16319A, and, in only one case, by a haplotype derived from the latter by an apparent reverse substitution (back mutations) at position 16227 [23, 24]. The other A10 haplotypes are completely absent. In the southwestern part of the area (including the forest-steppe zone of West Siberia, the Volga-Ural region, and adjacent territories in the south), the root haplotype of A10a (16223T, 16227C, 16230G, 16290T, 16311C, 16319A) predominates, and there are several divergent A10a lineages without C16256T transition [3, 20, 24–26] (S3 Table). Only the root A10a haplotype was found in the gene pool of Siberian Tatars, comprising modern populations of the West Siberian forest-steppe zone (including the Baraba region) [3]. Therefore, the Baraba forest-steppe is located in the center of the current distribution area of haplogroup A10, between its phylogenetically contrasting parts.

### Authenticity of the experimental results

We employed the experimental standards and measures against intra-laboratory contamination widely accepted by ancient DNA researchers [27–29] (see Materials and Methods). Direct and indirect evidence supports the authenticity of the ancient mtDNA sequences we obtained, as follows: for each individual under study, the mtDNA sequences obtained from multiple extracts, as well as from repeated PCRs of the same extracts, were identical; the data obtained for different parts of the mtDNA (HVR I, sites in the coding part) were consistent and can be unambiguously interpreted phylogenetically; none of the ancient sequences matched the sequences of the researchers (geneticists, anthropologists) who were in contact with the remains before or during the paleogenetic studies (among these researchers there were no carriers of haplogroup A or other East Eurasian mtDNA haplogroups); the results obtained from the different parts of the skeletons were identical in all cases; the probability of total contamination of the skeletons by an archaeologist or by a person not involved in the research is unlikely, especially considering the extremely low frequency of the A10 haplogroup in the gene pools of modern Eurasian populations; reconstruction of the consensus sequence based on the sequencing of several clones (S2 Table) eliminates the possible influence of miscoding lesions or random errors during PCR on the final data.

The following circumstances indicate that ancient samples were not cross-contaminated: experiments with haplogroup A10 samples dated to different periods of the Bronze Age were performed at different times with long time intervals between them; all series of samples include a large variety of lineages belonging to different East and West Eurasian haplogroups, in addition to the lineages of haplogroup A10 described in this paper (these data are being prepared for publication, see [12, 14] for some preliminary results); and five lineages of A10 haplogroup with different HVR I haplotypes were present among the ancient mtDNA samples.

Sex determination and autosomal STR analysis were successfully carried out for two samples (other samples were not analyzed) (S3 Table). The results of genetic sex determination are in agreement with anthropological data. The STR profiles indicate that the DNA samples studied belong to different individuals. The ancient STR profiles did not match the profiles of the geneticists conducting the experiments.

In conclusion, we confirm the authenticity of the ancient human mtDNA sequences presented here.

## Discussion

Our data on ancient mtDNA are particularly significant for the reconstruction of the development of the genetic and phenotypic specificity of West Siberian indigenous human populations. Based on data from physical anthropology, several modern indigenous populations from West Siberia were attributed to the Uralic (or West Siberian) anthropological type [1, 5], which is characterized by a complex combination of craniometric features typical of western Eurasian and eastern Eurasian populations. Undoubtedly, interactions between genetically and phenotypically contrasting western and eastern Eurasian groups were crucial factors in the evolution of West Siberian indigenous populations [30]. Archaeological data suggest that such interactions were quite intensive, at least from the beginning of the Middle Bronze Age (the beginning of II millennium BC). The significance of such interactions is confirmed by modern mtDNA data: studies of the mtDNA gene pool of modern indigenous populations from the northern (taiga and tundra zone) and southern (steppe and forest-steppe zone) parts of West Siberia revealed its mixed structure and the presence of mtDNA clusters of western and eastern Eurasian origin in different ratios [3, 23, 25, 31].

However, it was argued that the craniometric specificity of modern West Siberian populations could not be completely explained by such genetic interactions [1, 2]. It was suggested that an ancient indigenous human group with undifferentiated anthropological characteristics underwent a long-term independent evolution in the region, and then contributed to the formation of the specificity of West Siberian population [1, 6]. Later, this group was subjected to the strong genetic influence of the eastern Eurasian and western Eurasian groups that had migrated to the region since the Middle Bronze Age and thus it was hypothesized that this series of events determined the gene pool structure of the modern indigenous populations.

From the genetic point of view, this hypothesis can be confirmed by the presence, in the gene pool of indigenous West Siberian populations, of components with autochthonous origin or/and long-term evolution in the region. These components may be represented by some subclusters of Y-chromosome N1b haplogroup that show signs of autochthonous origin in the ancestral gene pool of Uralic-speaking human groups [32].

To our knowledge, no published study to date includes evidence for indigenous components at the clusters or subclusters level in the mtDNA gene pool of modern West Siberian aboriginal populations, including Ugric, Samoyed and Turkic-speaking populations, but with the exception of a few lineages within U4 haplogroup [33].

In the light of our ancient DNA data, we suggest that the mtDNA haplogroup A10 is an autochthonous component of the gene pool of West Siberian indigenous populations, and that this component underwent a long evolution in West Siberia before the arrival in the region of genetically contrasting western Eurasian and eastern Eurasian groups.

Considering the specificity of human population history in the region, a genetic component potentially indicating the contribution of the ancient autochthonous population to the gene pool structure of modern West Siberian populations must meet the following criteria:

1. It should be distributed in the ancient population within a territory that corresponds to a potential region of development of the population under consideration. The area of its distribution must be large enough to have an impact on the development of populations in the larger territory.
2. It must have been present in the gene pool of West Siberian populations for a long time, at least since before large-scale migrations to the region and genetic interactions with newly arrived groups.
3. The cluster must have undergone several stages of its evolution in the region.
4. It must show a specific distribution in modern populations evolved in the region for a long time. The variability and frequency in the gene pool of the component is highly likely to be, but not necessarily, different in the modern and in the ancient populations, as a result of recent influence of genetically contrasting groups that migrated to the region or owing to an effect of random genetic drift.

In the context of this research, it is of particular significance that the supposed genesis center of the phenotypic specificity of West Siberian populations (Ural (or West Siberian) anthropological type genesis) is placed by anthropologists in the southern taiga and the forest-steppe zones of West Siberia [1, 5], including the northern part of the Baraba forest-steppe, from which the paleoanthropological materials under study were obtained [2].

Moreover, the earliest human groups of the West Siberian forest-steppe for which paleoanthropological materials are available are dated to the Neolithic period, and Baraba populations of the subsequent Early Metal Age and Bronze Age (including populations of Ust-Tartas, Odinovo and Krotovo cultures we have studied) already showed features of West Siberian anthropological type [2, 11]. Therefore, the Bronze Age populations we studied are highly likely to have been involved in the early stages of genesis of West Siberian indigenous populations and their genetic (and phenotypic) specificity.

Archaeological and anthropological data show that the local ancient Baraba populations under study were not strongly isolated, either culturally or genetically from the populations of neighboring forest-steppe, southern taiga and steppe areas [13]. The individuals we identified with the A10 haplogroup lineages represent the major Bronze Age cultural groups that occupied vast areas within West Siberia (Table 1).

The presence of several HVRI haplotypes among the samples and the significant differences in chronology of the populations under study suggest that close relationships between the ancient individuals studied are unlikely to have influenced the results (see details in S1 File). These facts suggest that the observed pattern of A10 presence and variability is not a local trait of the ancient populations studied, but is a general characteristic of the Bronze Age populations of the West Siberian forest-steppe belt and adjacent south-taiga and northern steppe regions.

The paleogenetic approach allowed us to detect, without doubt, the presence of a genetic lineage in the gene pools of the ancient populations, and to determine a minimal age for its occurrence in the region, by analyzing samples with precise geographical and chronological attribution. A10 lineages were detected in all Bronze Age populations from the Baraba forest-steppe. The most ancient West Siberian population under study in which A10 haplogroup lineage was found is the Ust-Tartas culture population (Neolithic populations have not been studied to date). The A10 lineage identified in two Ust-Tartas samples differs from the A10 root haplotype by substitution at the 16148 position, so we can conclude not only that mtDNA haplogroup A10 was present in the mtDNA gene pool of the population of the West Siberian forest-steppe zone, but also that it had already passed the initial stage of diversification, resulting in the appearance of A10 lineages diverged from the root haplotype at least ~5500–6000 years ago. Clearly, this haplogroup originated and the initial stages of its diversification occurred in earlier periods: in the Neolithic (i.e., Early Holocene), or even in the Late Pleistocene. This assumption is indirectly confirmed by the high degree of continuity between the Neolithic population of the region (~8000–7000 years of age) and the populations of the Early Metal Age and the Early and Middle Bronze Age (6000–4000 years of age), revealed by physical anthropological methods [2].

The earliest archaeological materials (from a few sites with scant traces of stone tool use) found so far in the well-studied Baraba forest-steppe are only 13,000–14,000 years old, i.e., they date from the final Pleistocene (and post-Last Glacial Maximum [LGM]) period [34] (though rare archaeological finds indicate an earlier presence of a small number of people in other parts of forest-steppe and taiga zones of West Siberia in the Late Paleolithic [35–37]). Extremely small numbers of people probably existed in the region, possibly not permanently, primarily due to severe climatic conditions during the LGM and excessive watering of this territory during the early post-LGM period. The permanent presence in the region of a relatively large population has been documented archaeologically only since the Neolithic period ~8000 years ago [38].

Therefore, our data indicate the presence of haplogroup A10 lineages in the ancient populations of the West Siberian forest-steppe zone for most of the period of intensive inhabitation of the region. A substantial part of this period (until the beginning of II millennium BC) preceded the first significant migrations to the region and the interaction there of genetically contrasting human groups [12].

Phylogeographic analyses to study early population processes have been conducted for several other subclusters of mtDNA haplogroup A: the evolution of A2 cluster is related to early settlement of North-East Asia in the Late Paleolithic, and the subsequent settlement of America [39, 40]; the appearance of cluster A11 in Tibet dates back to the post-LGM period [41, 42]. Subsequent genetic isolation of human populations in these regions has led to the preservation of these components with high frequencies and diversity [39–41].

The origin and initial stages of haplogroup A10 diversification were probably associated with early groups that infiltrated West Siberia in the final Pleistocene (post-LGM) or early Holocene, and then evolved independently in the region for a long period, allowing the formation of early features of regional population`s gene pool. Under the influence of genetically contrasting groups from western and eastern Eurasia, intensively migrated into the region from the first half of the II millennium BC (Andronovo, Xuongnu (Xun), Turkic, Mongolian, etc.), the role in the gene pool of the ancient indigenous components must have been greatly reduced, or they disappeared completely.

The specificity of the A10 mtDNA haplogroup’s distribution in ancient and modern Eurasian populations is in good agreement with this scenario. The overall distribution of the A10 haplogroup in modern populations of Eurasia corresponds with its origin and/or evolution in West Siberia. A10 lineages are present in gene pools of modern Ugric, Samoyed and Turkic-speaking groups from the central region of northern Eurasia (Fig 3). The south of West Siberia is located in the center of the whole A10 area, and it is also the place where the ancient ancestors of some of these ethnic groups could interact (in particular, southern proto-Ugric groups and ancient Turkic-speaking populations interacted in the West Siberian forest-steppe zone) [1, 43]. Independent evolution of ancestral groups in geographically remote regions could lead to the formation of phylogenetically contrasting (in terms of A10 lineages) populations within the general area (as we found—see Results). Moreover, A10 lineages are absent in many modern populations of West Siberia and adjacent regions. The overall frequency and diversity (number of diversification vector from the root lineage) of haplogroup A10 lineages are significantly lower in the modern indigenous populations of the main A10 area in Eurasia than in the Bronze Age populations of West Siberia under study (see S1 File). The variability of A10 lineages in West Siberian Bronze Age populations represents different stages of evolution and several diversification vectors of the A10 cluster, whereas all of the lineages identified in modern populations within the main A10 area belong to the subcluster A10a. As for A10 lineages found outside the main area, they are either phylogeographically unexplained (Italian Alps) [21], or are likely to come from the main area (Afghanistan; A10 lineages were probably introduced with migration of Indo-Iranian speaking nomads from northern steppes) [22]. The significant decline in diversity of the haplogroup A10 in modern populations is also evidenced by the fact that the diversity of A10 lineages detected (n = 5) in ancient populations of a small region in Baraba forest-steppe (96 individuals) is equivalent (in quantitative but not qualitative terms) to their total diversity (n = 7) in all modern populations inhabiting the area of this haplogroup studied to date (thousands individuals). This also demonstrates that the structure of the gene pool observed in modern populations does not always reflect the full set of genetic components that characterize the genetic history of populations in the region, especially those characterizing their early genetic history.

Therefore, haplogroup A10 has all the features expected for the indigenous component of the mtDNA gene pool of the aboriginal populations of West Siberia. It is highly probable that West Siberia is the place of origin of A10 or, at any rate, the place of its long-term evolution and diversification; thus the mtDNA haplogroup A10 could be one of the genetic markers of the ancient autochthonous population that has contributed to the early stages of specificity formation of West Siberian indigenous populations, independently of the subsequent influence of genetically contrasting groups.

We expect that there were other autochthonous mtDNA markers in the regional population’s gene pool. They may be present as rare variants or be almost absent in modern populations of the region under study. Our study demonstrates the effectiveness of our approach to research on assessing the significance of such elements in the gene pool of the West Siberian populations. It is necessary to continue studying the ancient populations of West Siberia and adjacent areas from the Neolithic period to the Middle Ages to obtain an objective reconstruction of the genetic history of this region. We have already begun to implement such a large-scale project.

## Supporting Information

S1 Table PCR-primers used for amplification of mtDNA fragments. (DOC)

S2 Table Alignment of mtDNA HVR I sequences. (PPT)

S3 Table Autosomal STR allelic profiles of the Bronze Age individuals from Baraba region. (DOC)

S1 File Distribution of A10 haplogroup lineages in modern human populations of Eurasia and ancient West Siberian human groups. (DOC)

S2 File Caracteristics of cultural groups, archaeological sites and anthropological samples, analyzed in this work. (DOC)
