# Supplementary material for: MtDNA Haplogroup A10 Lineages in Bronze Age Samples Suggest That Ancient Autochthonous Human Groups Contributed to the Specificity of the Indigenous West Siberian Population
Source: PLoS One. 2015 May 7;10(5):e0127182. doi: 10.1371/journal.pone.0127182 (PMC4423966; doi:10.1371/journal.pone.0127182)
Supplement: S1 Table — (DOC) [file pone.0127182.s001.doc]

**S1 Table**. PCR-primers used for amplification of mtDNA fragments.

| Fragment (position) of mtDNA (without primers) | PCR primers | Reference |
| --- | --- | --- |
| 15997-16141 | L15996/16142 | [5] |
| 16118-16232 | L16117/H16233 | [5] |
| 16210-16347 | L16209/H16348 | [5] |
| 16288-16409 | L16287/H16410 | [5] |
| 636-376 (663) | L635/H677 | [15] |
