# Supplementary material for: MtDNA Haplogroup A10 Lineages in Bronze Age Samples Suggest That Ancient Autochthonous Human Groups Contributed to the Specificity of the Indigenous West Siberian Population
Source: PLoS One. 2015 May 7;10(5):e0127182. doi: 10.1371/journal.pone.0127182 (PMC4423966; doi:10.1371/journal.pone.0127182)
Supplement: S2 Table — (PPT) [file pone.0127182.s002.ppt]

## Slide 1
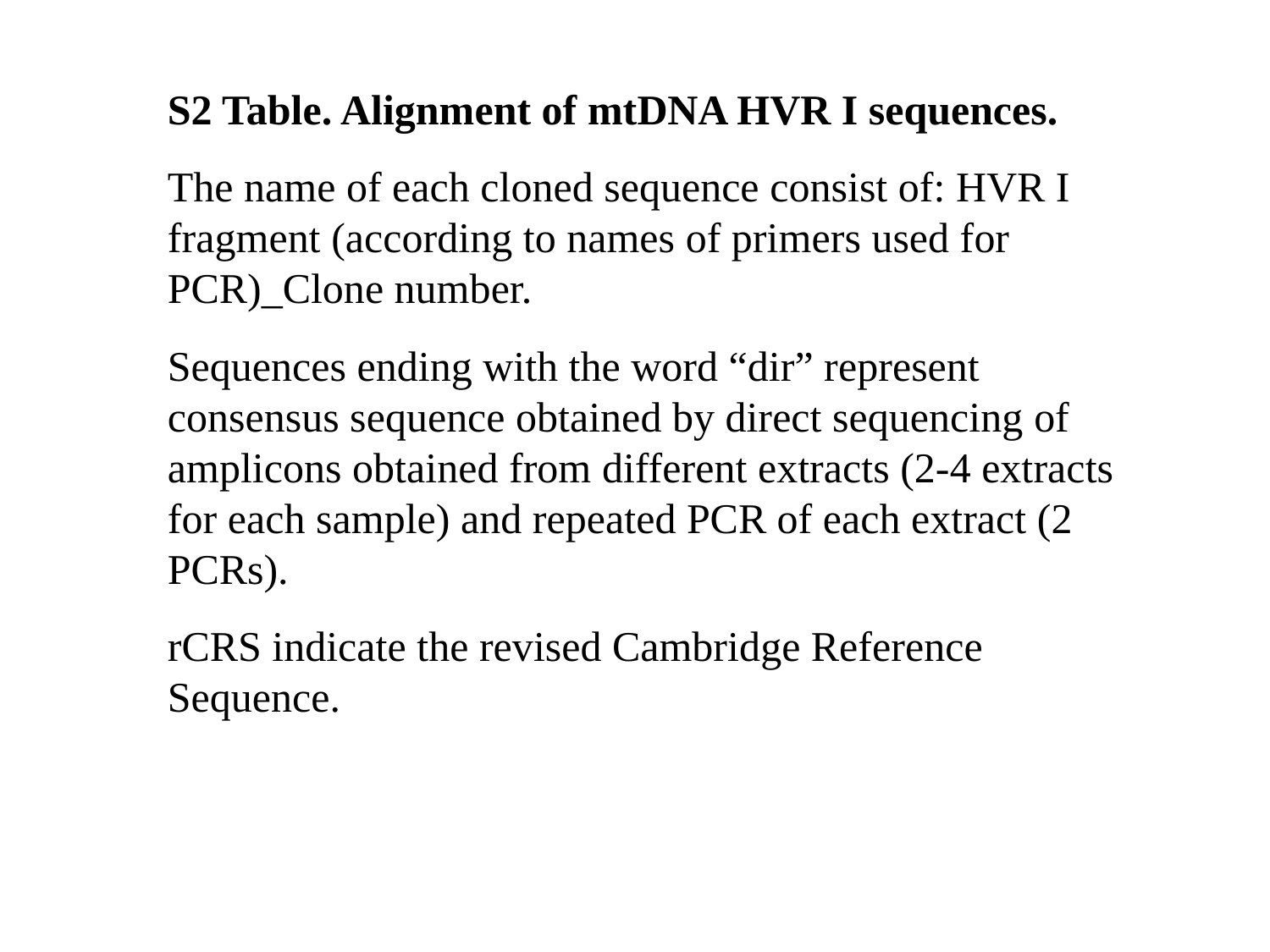

S2 Table. Alignment of mtDNA HVR I sequences.
The name of each cloned sequence consist of: HVR I fragment (according to names of primers used for PCR)_Clone number.
Sequences ending with the word “dir” represent consensus sequence obtained by direct sequencing of amplicons obtained from different extracts (2-4 extracts for each sample) and repeated PCR of each extract (2 PCRs).
rCRS indicate the revised Cambridge Reference Sequence.

## Slide 2
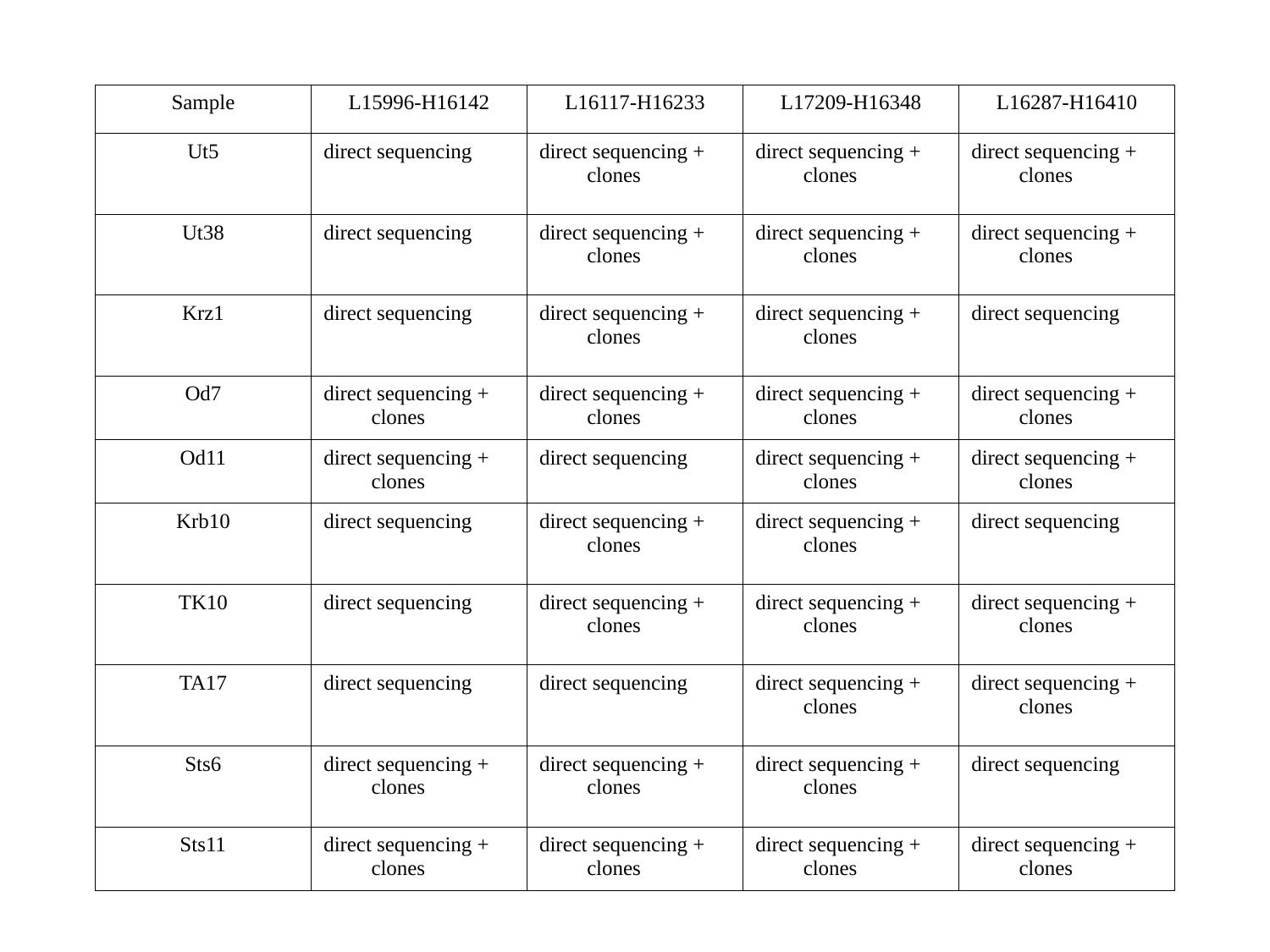

| Sample | L15996-H16142 | L16117-H16233 | L17209-H16348 | L16287-H16410 |
| --- | --- | --- | --- | --- |
| Ut5 | direct sequencing | direct sequencing + clones | direct sequencing + clones | direct sequencing + clones |
| Ut38 | direct sequencing | direct sequencing + clones | direct sequencing + clones | direct sequencing + clones |
| Krz1 | direct sequencing | direct sequencing + clones | direct sequencing + clones | direct sequencing |
| Od7 | direct sequencing + clones | direct sequencing + clones | direct sequencing + clones | direct sequencing + clones |
| Od11 | direct sequencing + clones | direct sequencing | direct sequencing + clones | direct sequencing + clones |
| Krb10 | direct sequencing | direct sequencing + clones | direct sequencing + clones | direct sequencing |
| TK10 | direct sequencing | direct sequencing + clones | direct sequencing + clones | direct sequencing + clones |
| TA17 | direct sequencing | direct sequencing | direct sequencing + clones | direct sequencing + clones |
| Sts6 | direct sequencing + clones | direct sequencing + clones | direct sequencing + clones | direct sequencing |
| Sts11 | direct sequencing + clones | direct sequencing + clones | direct sequencing + clones | direct sequencing + clones |

## Slide 3
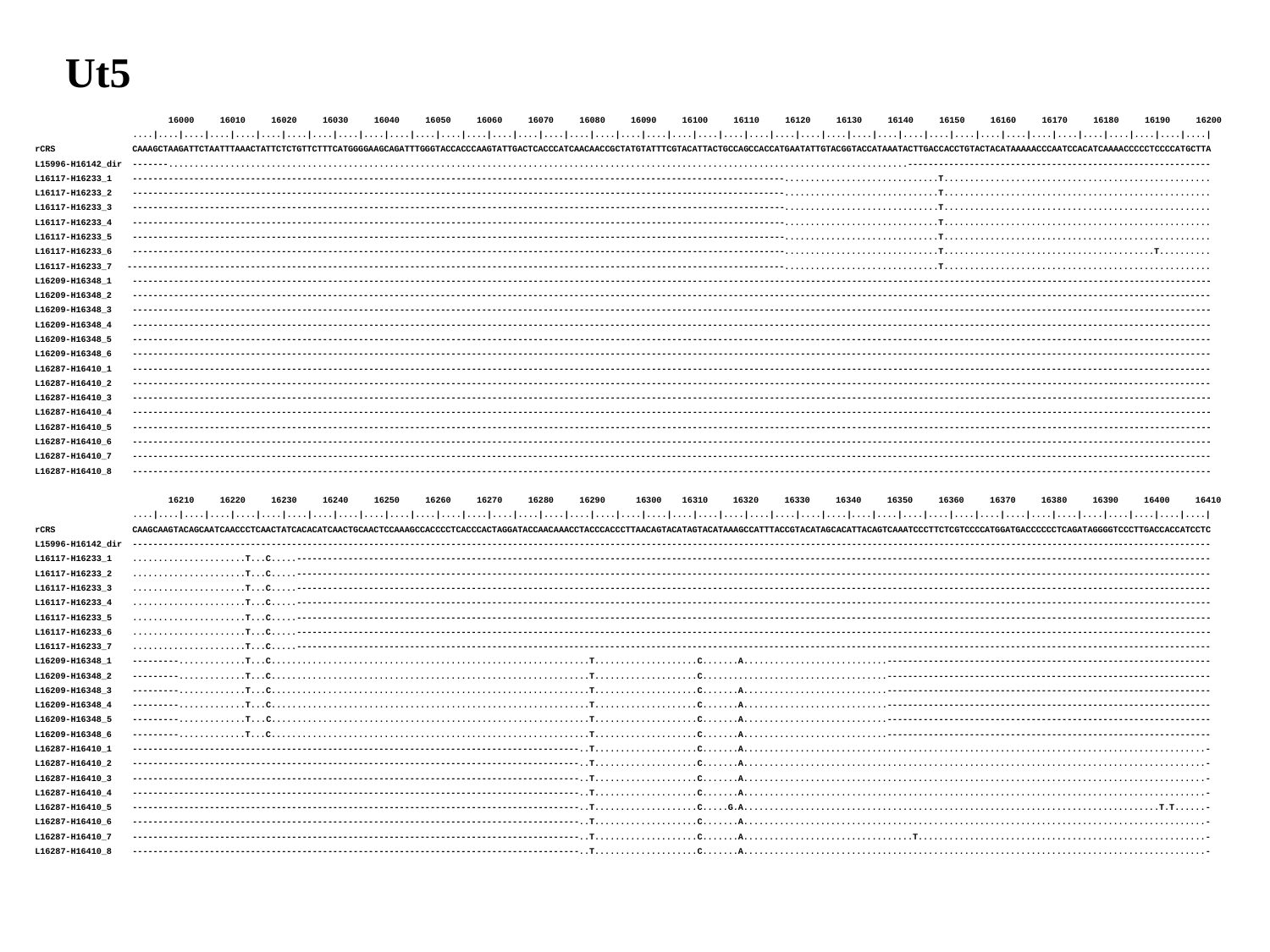

Ut5

## Slide 4
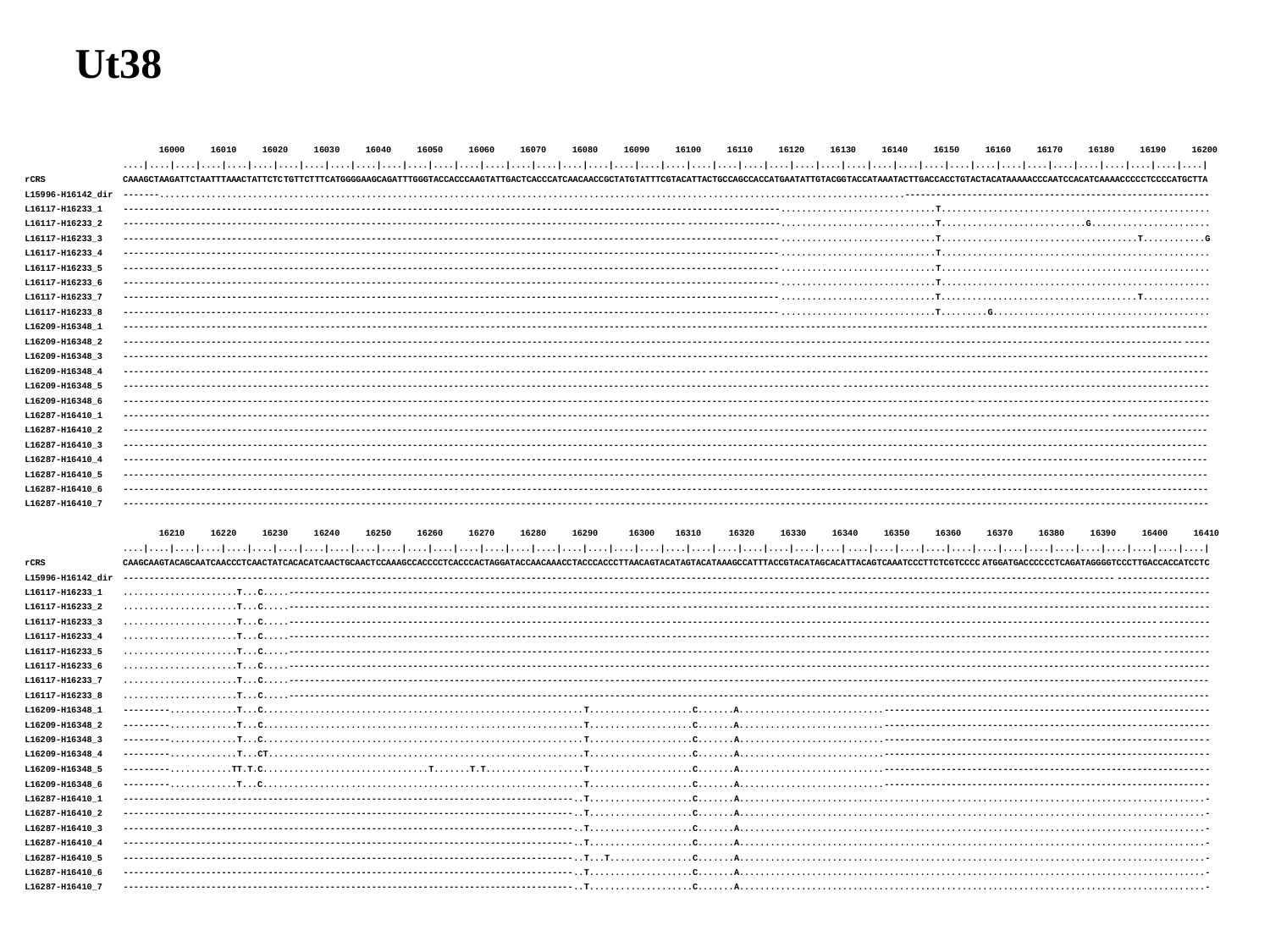

Ut38

## Slide 5
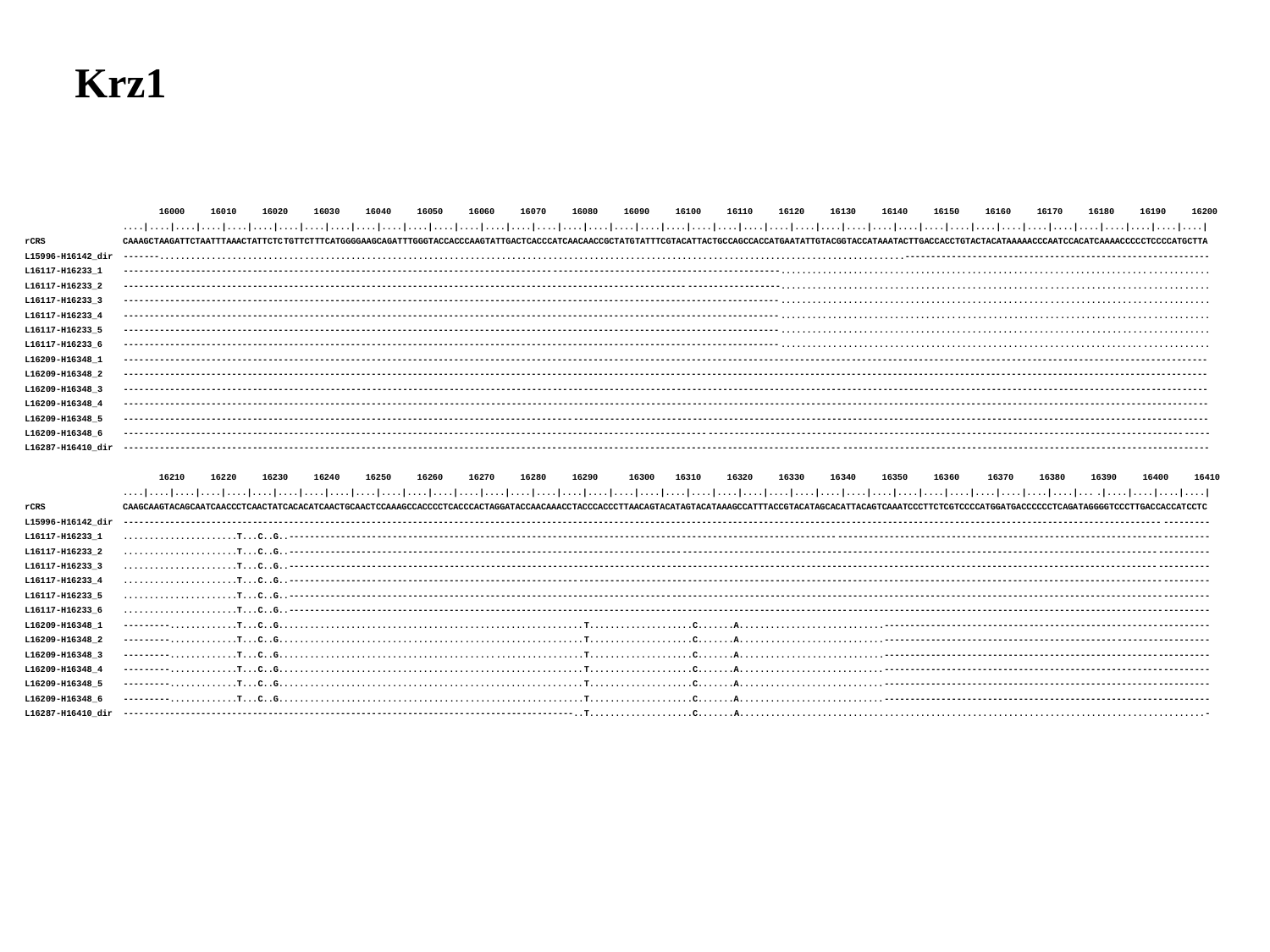

Krz1

## Slide 6
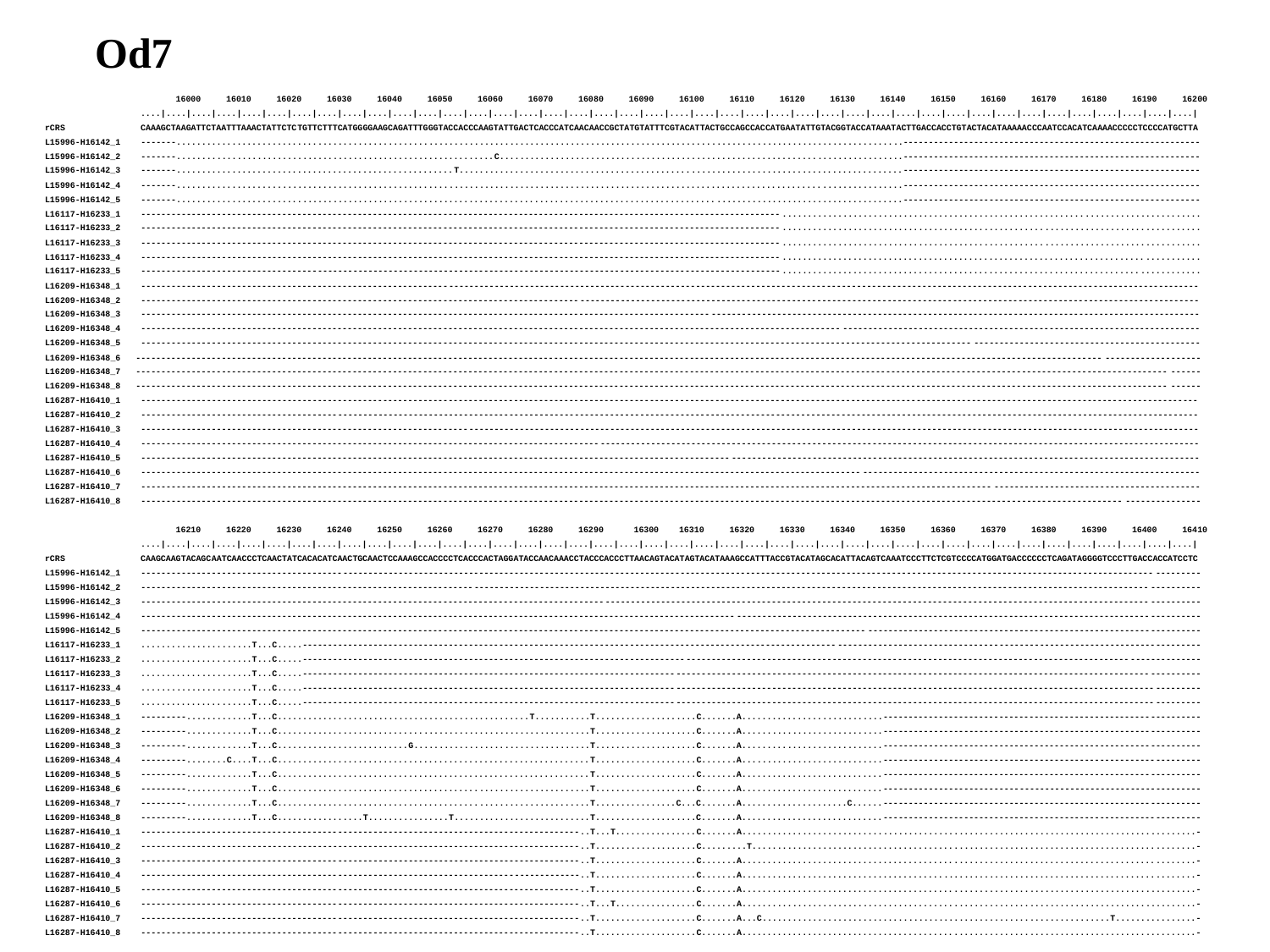

Od7

## Slide 7
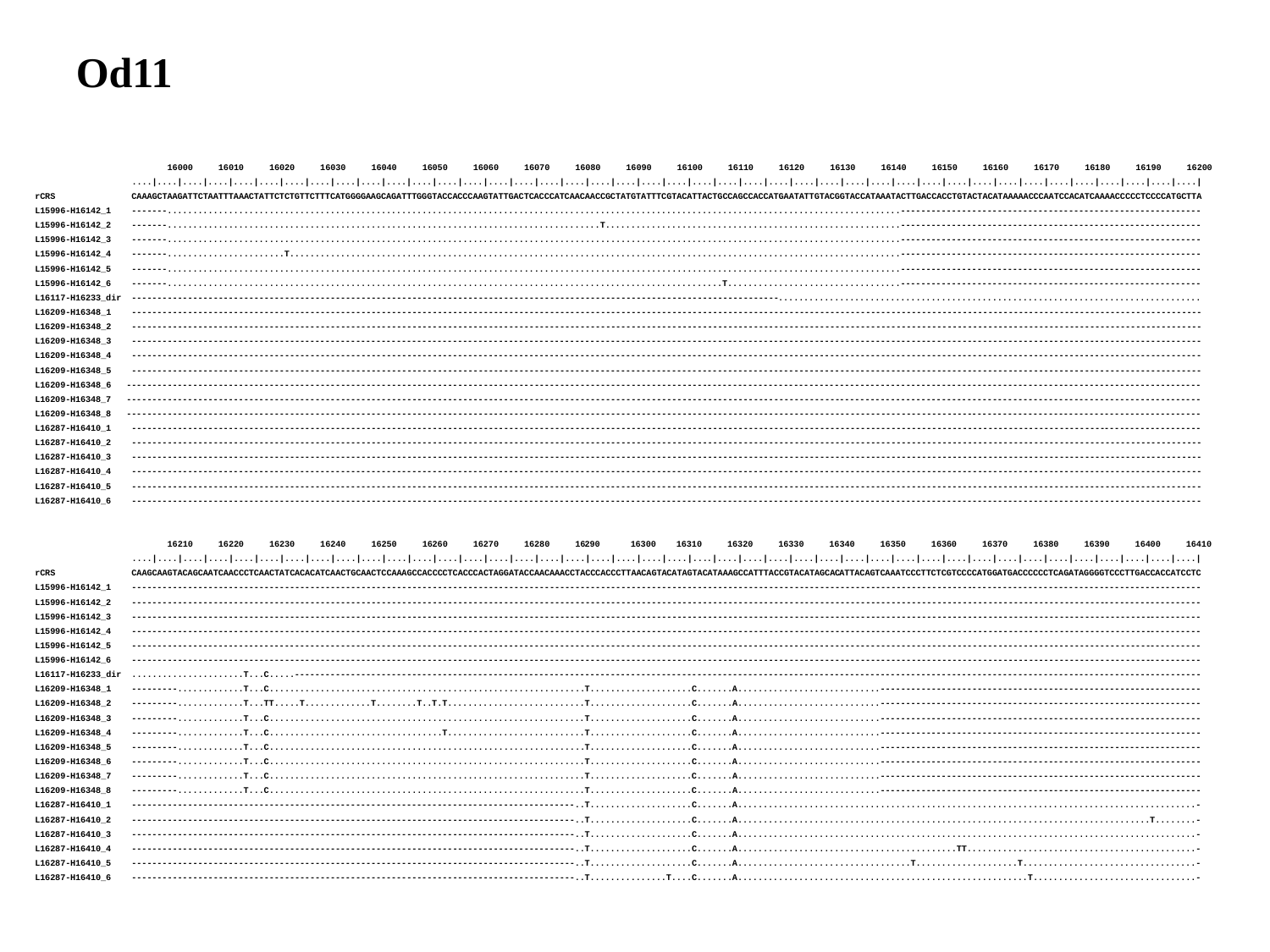

Od11

## Slide 8
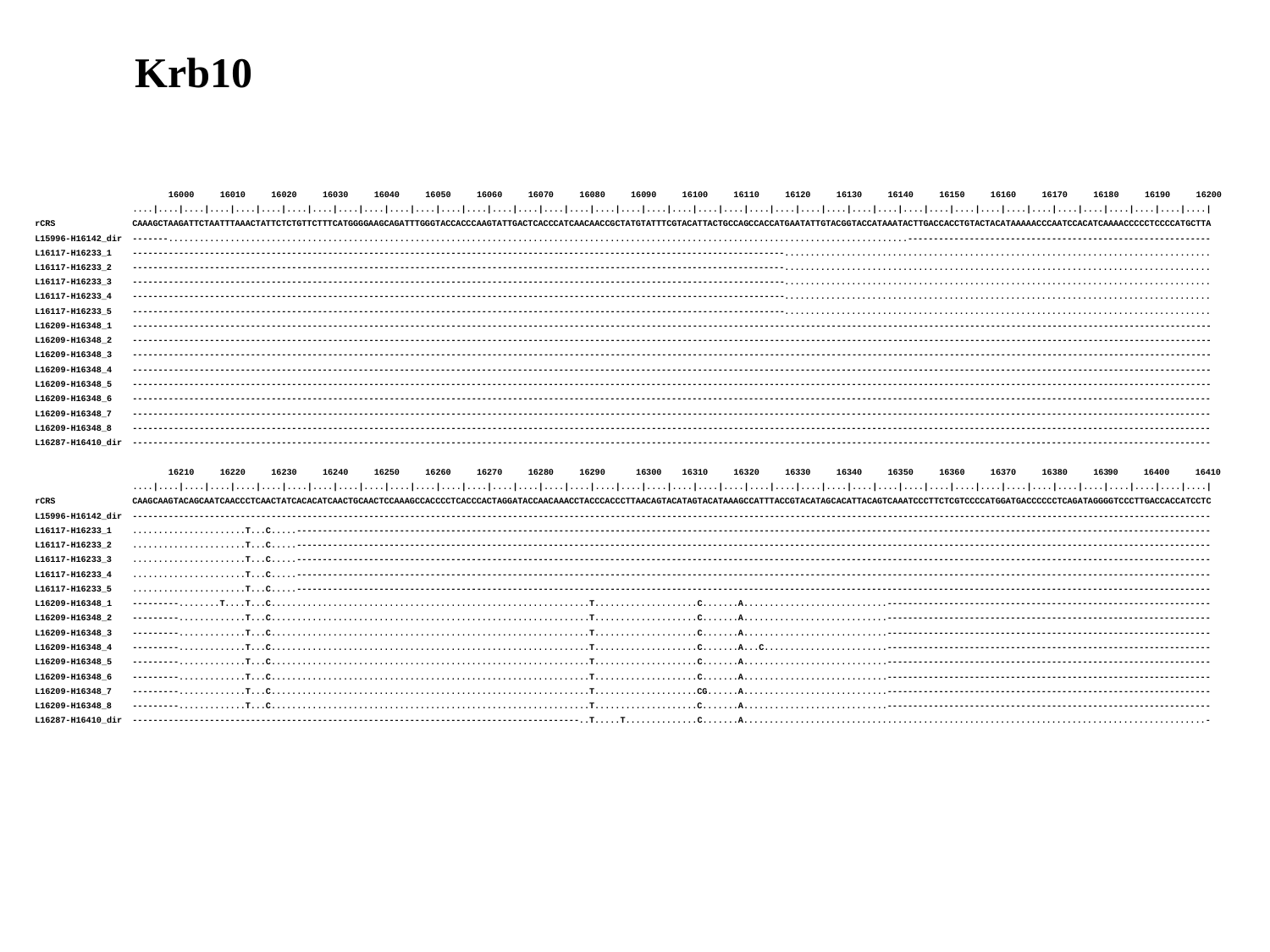

Krb10

## Slide 9
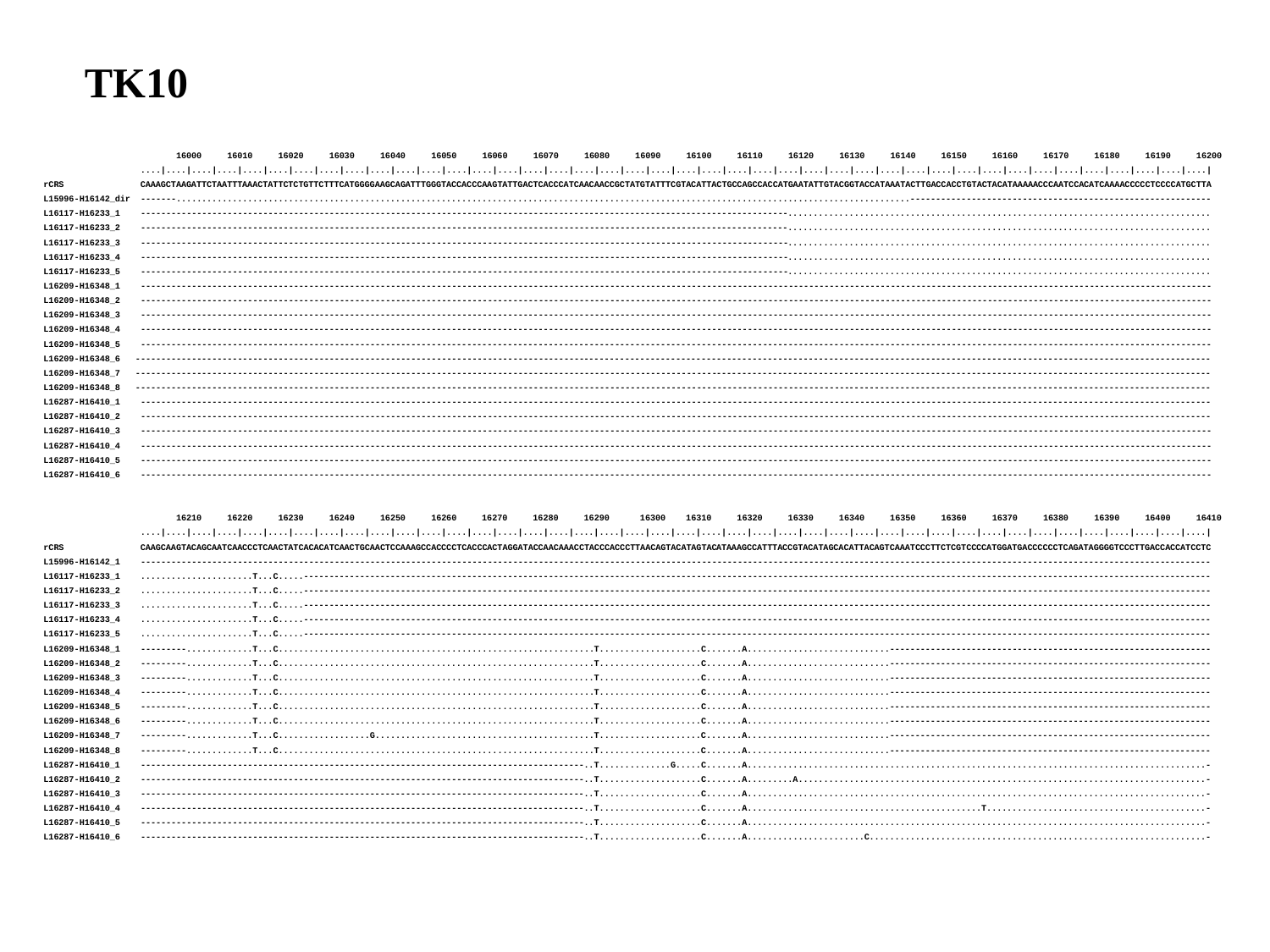

TK10

## Slide 10
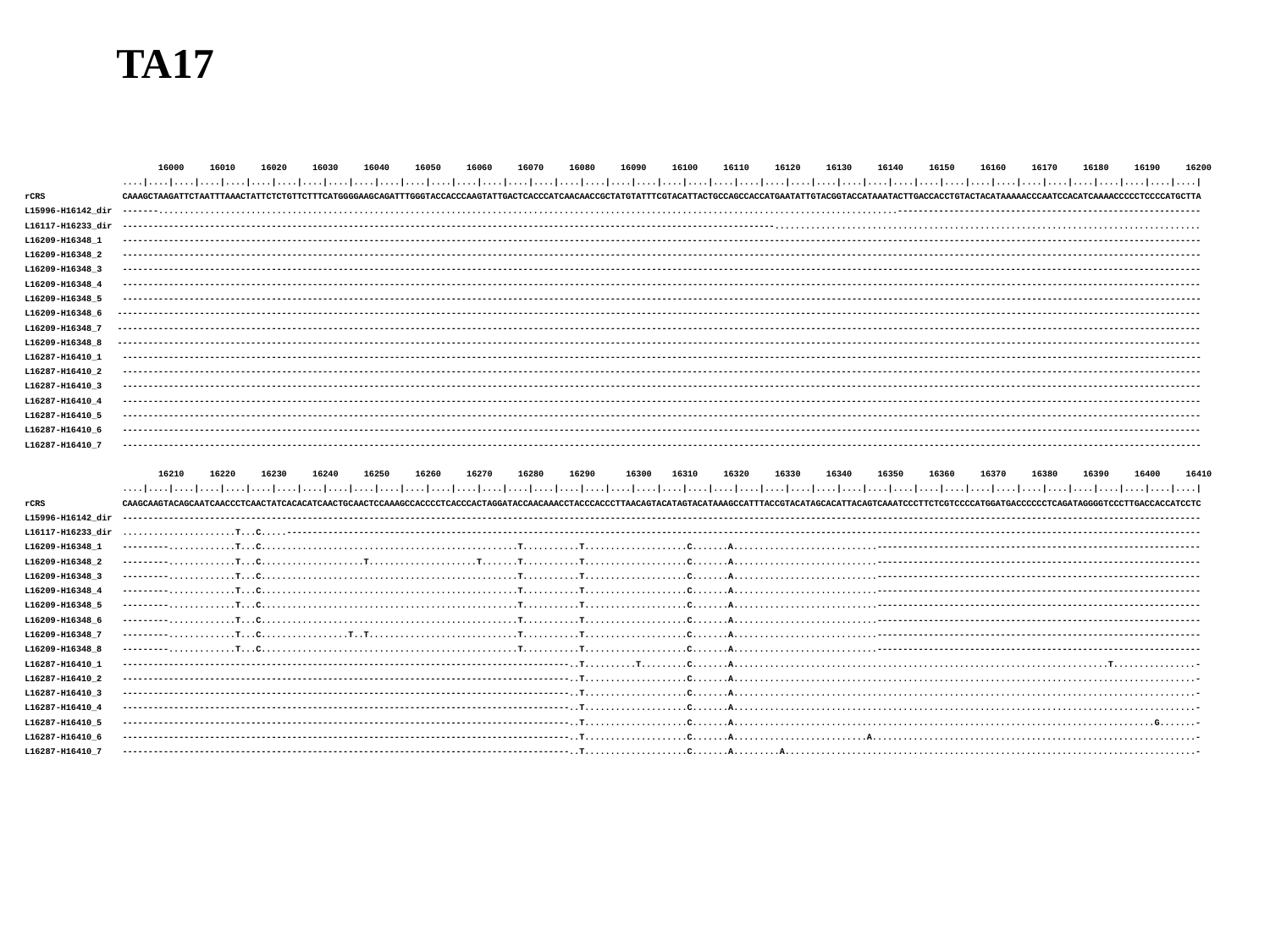

TA17

## Slide 11
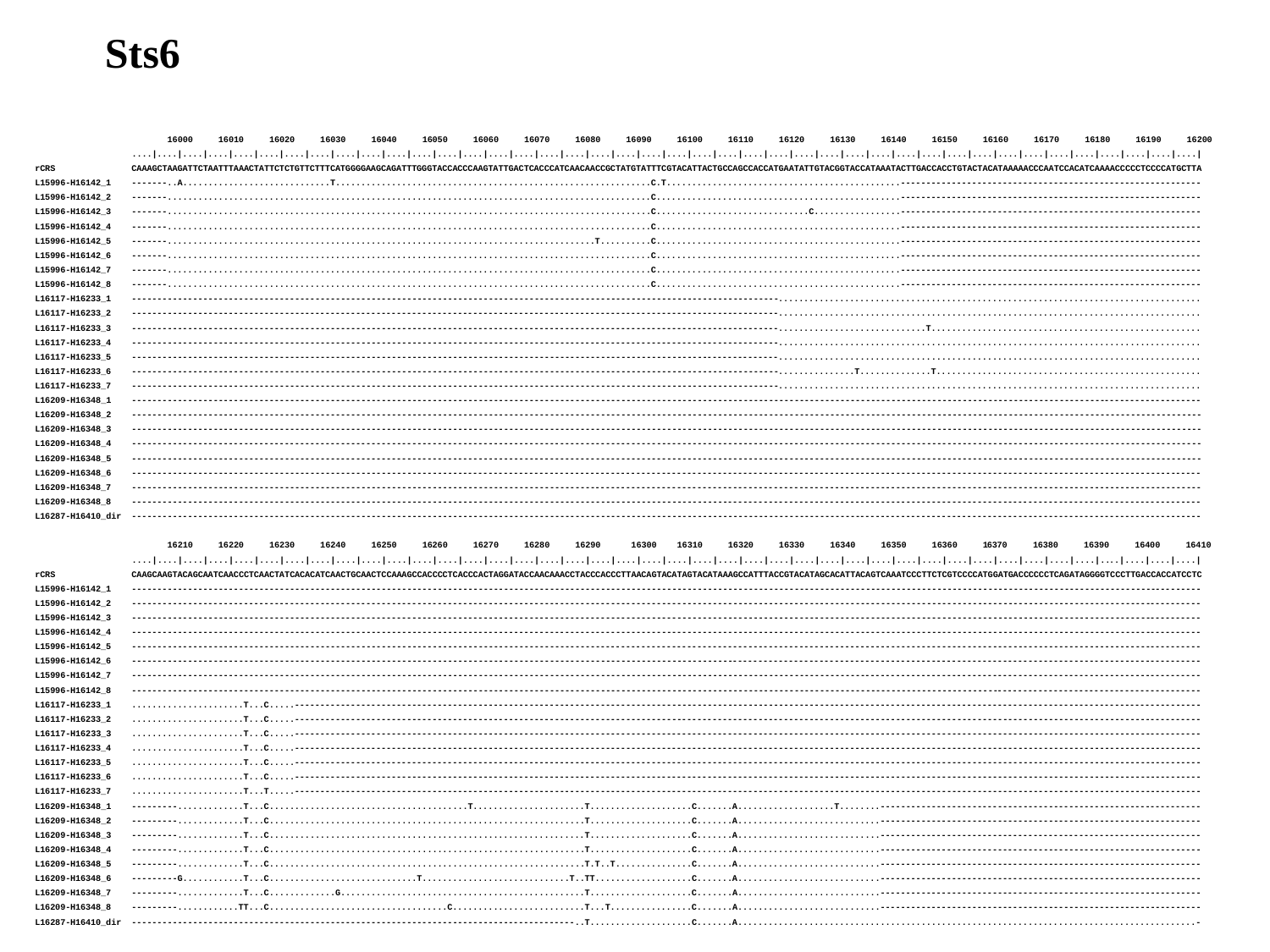

Sts6

## Slide 12
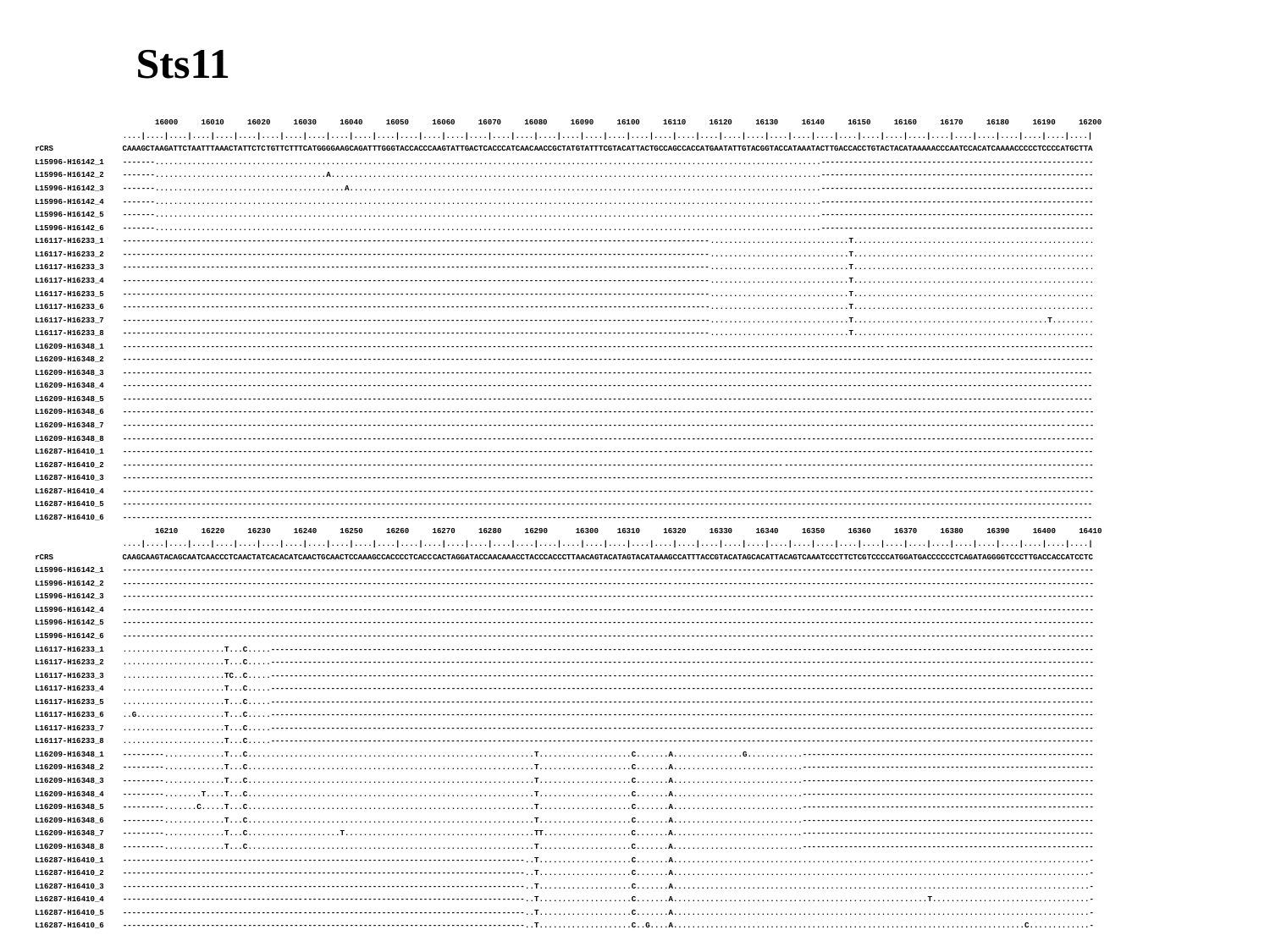

Sts11
