# Supplementary material for: MtDNA Haplogroup A10 Lineages in Bronze Age Samples Suggest That Ancient Autochthonous Human Groups Contributed to the Specificity of the Indigenous West Siberian Population
Source: PLoS One. 2015 May 7;10(5):e0127182. doi: 10.1371/journal.pone.0127182 (PMC4423966; doi:10.1371/journal.pone.0127182)
Supplement: S3 Table — (DOC) [file pone.0127182.s003.doc]

**S3 Table. Autosomal STR allelic profiles of the Bronze Age individuals from Baraba region.**

| Sample | Sex | D3S1358 | vWA | FGA | D8S1179 | D21S11 | D18S51 | D5S818 | D13S317 | D7S820 |
| --- | --- | --- | --- | --- | --- | --- | --- | --- | --- | --- |
| Ut38 | XX | 15/16 | 16/18 | - | 12/14 | 30/30 | 14/14 | 12/13 | 10/10 | 11/11 |
| Od11 | XY | 16/17 | 17/17 | 19/19 | 13/14 | 30/30 | - | 7/11 | - | - |

STR genotyping was performed several times for each individual (at least two times for each of the two extracts, i.e. a total of at least 4 repeats of STR amplification for the individual). In the final version of the results were only alleles stably supported by the analysis of repeated PCR. We are aware that some homozygosity cases may be due to inability to amplify longer allelic variants, especially for STR-loci having a greater length of amplicons (much greater than 200 bp, in our case it's probably for the loci FGA, D8S51, D7S820 ). Homozygous status is set when the amplification product of the second allele never been identified. Comparison with the corresponding profiles STR-loci laboratory staff used as an additional control to exclude intra-laboratory contamination.
