# Supplementary material for: MtDNA Haplogroup A10 Lineages in Bronze Age Samples Suggest That Ancient Autochthonous Human Groups Contributed to the Specificity of the Indigenous West Siberian Population
Source: PLoS One. 2015 May 7;10(5):e0127182. doi: 10.1371/journal.pone.0127182 (PMC4423966; doi:10.1371/journal.pone.0127182)
Supplement: S1 File — (DOC) [file pone.0127182.s004.doc]

**S1 File.**

**Distribution of A10 haplogroup lineages in modern human populations of Eurasia.**

| Haplotype of mtDNA HVR I | Population (number of individuals/whole number of studied individuals in the group) | Reference | Subcluster of A10 |
| --- | --- | --- | --- |
| 16223T, 16227C, 16230G, 16290T, 16311C, 16319A | Siberian tatars (2/218) | [23] | A10a |
|  | Tatars (Volga-Ural region) (5/197) | [18] | A10a |
|  | Tatars (Volga-Ural region) (4/228) | [24] | A10a |
|  | Bashkirs (1/221) | [24] | A10a |
|  | Chuvash (2/55) | [24] | A10a |
|  | Mari (1/136) | [24] | A10a |
|  | Nogays (2/206) | [25] | A10a |
|  | Tajiks (1/38) | [22] | A10a |
| 16223T, 16227C, 16230G, 16256T, 16290T, 16311C, 16319A | Nganasans (5/107) | [22] | A10a |
|  | Dolgans (3/157) | [22] | A10a |
|  | Mansi (1/98) | [21] | A10a |
| 16223T, 16230G, 16256T, 16290T, 16311C, 16319A | Evenks (1/324) | [22] | A10a |
| 16221T, 16223T, 16227С, 16230G, 16290T, 16311C, 16319A | Bashkirs (1/221) | [24] | A10a |
| 16223T, 16227С, 16230G, 16290T, 16311C,16317T, 16319A | Mari (1/136) | [24] | A10a |
| 16129A, 16223T, 16227С, 16290T, 16311C, 16319A | Population of Italian Alps (1/393) | [19] | A10* |
| 16223T, 16227С, 16290T, 16311C, 16319A | Afghanistan Hazara (1/78) | [20] | A10* |
|  | Afghanistan Uzbek (1/127) | [20] | A10* |

**List of modern human populations of Eurasia used for phylogeographic analysis:**

West Siberia: Khants (210), Komi (78) (Gubina et al. 2005), Mansi (98) (Derbeneva *et al.*, 2002), Mansi (63), Khants (106) (Pimenoff et al., 2008), Siberian Tatars (218) (Naumova et al., 2008), Kets (38) (Derbeneva et al., 2002), Nganasans (107) (Tamm et al., 2007);

South Siberia: Altaians (110), Khakassians (53), Buryats (91), Soyots (30), Todzhins (48), Tuvinians (90), Tofalars (58) (Derenko *et al.*, 2003), Buryats (295), Khamnigans (99), Tuvinians (105), Shors (82), Khakassians (57), Altaians-Kizhi (90), Telenghits (71), Teleuts (53); Tubalars (72), Tuvinians (95), Buryats (25), Tofalars (46) (Starikovskaya et al., 2005); Altaian Kazakhs (237) (Gokcumen et al., 2008).

East and North-Eastern Siberia, the Far East: Evens (Derenko, Shields, 1997), Yakuts (191) (Fedorov et al., 2003) (117) (Pakendorf et al., 2003); Nentsi (58) (Saillard et al., 1999), Evenks (71) (Starikovskaya et al., 2005); East Evenks (45), West Evenks (73), Yakuts (36) (Derenko et al., 2007) nivkhs (57) (Torroni et al., 1993b), Oroks (61) (Bermisheva et al., 2005) ; Siberian Eskimos (126), Chukchi (182), Yukagirs (132) (Volodko et al., 2008); Negidal (33), Ulchi (87), Nivkhi (56), Udegey (46) (Starikovskaya et al., 2005);

Volga-Ural Region: Komi-permians (74), Komi-ziryans (62), Mari (136), Udmurt (101), Mordvinians (102), Tatars (228), Bashkirs (221), Chuvash (55) (Bermisheva et al., 2002); Tatars (197) (Malyarchuk et al., 2009); Kalmyks (110) (Derenko et al., 2007).

Central and Middle Asia: Kazakhs (Kazakhstan) (52) (Comas et al., 1998), Kazakhs (Xinjiang) (30) (Yao et al., 2000), Kyrgyz (92) (Comas et al., 1998) Uighurs (Kazakhstan) (55) (Comas et al., 1998), Uyghurs (Xinjiang) (45) (Yao et al., 2000), Uzbeks (42), Turkmens (41), Kurds (Turkmenistan) (32) (Quintana-Murci et al. 2004), Mongolians (103) (Kolman et al., 1996), Mongolians (89) (Gokcumen et al., 2008); Afghanistan Hazara (78), Afghanistan Uzbek (127) (Di Cristofaro et al., 2013).

Southeast Asia: China - Han (410) – data collection from (Metspalu et al., 2004); the (35), Tibetans (41), well (30), Sali (30) (Yao et al., 2002).
India's population - the collection of data from work (Metspalu et al., 2004).

Middle East: Iranians (436) (Metspalu et al., 2004), Iraqis (116), the Syrians (69) (Richards et al., 2000), Turks (218) (Calafell et al., 1996; Comas et al. 1996; Richards et al., 2000), Kurds (Eastern Turkey) (53), (Richards et al., 2000), Palestinians (117) (Di Rienzo, Wilson, 1991; Richards et al., 2000), Nubian (80), the Egyptians (67) (Krings et al., 1999), Yemen Jews (43) (Di Rienzo, Wilson, 1991; Richards et al., 2000) Bedoons (29) (Di Rienzo, Wilson, 1991), Arabs in Saudi Arabia (120) (Abu-Amero et al., 2007), Druze (Israel) (45) (Macaulay et al., 1999);

Caucasus: Armenian (191), Azerbaijanis (48) (Richards et al. 2000), Georgians (45), Kurds (Tbilisi) (29) (Comas et al., 2000);

North Caucasus: Adygeys (50) (Macaulay et al., 1999), Nogays (206) (Bermisheva et al., 2004), the North Ossetians (114) (Macaulay et al., 1999).

Europe: South-Eastern Europe - Bulgaria (141) (Calafell et al., 1996; Richards et al., 2000), Romania (92) (Richards et al., 2000); Eastern Mediterranean - Greek (65) sarakatsani (Northern Greece) (60) (Richards et al., 2000), Albanians (42) (Belledi et al., 2000); Central Mediterranean - the Italians of Tuscany (49) (Francalacci et al., 1996; Torroni et al., 1998), the Italians from Rome (48), the Sicilians (90) (Richards et al., 2000), the population of Sardinia (115) (Di Rienzo, Wilson, 1991; Richards et al., 2000); Western Mediterranean - Portuguese (54) Spanish (71) (Corte-Real et al., 1996), the population of Galicia (92) (Salas et al., 1998); Alps - Swiss (70) (Pult et al., 1994), the southern Germans (Bayern) (49) (Richards et al., 1996), the Austrians (99) (Parson et al., 1998), Italian Alps (393) (Coia et al., 2012), North Central Europe - Poles (37), Czechs (83), the Germans (174) (Richards et al., 1996; Hofmann et al., 1997), Poles (436) (Malyarchuk et al., 2002), the Danes (38) (Richards et al., 1996, 2000); Scandinavia - Swedish (32) (Sajantila et al., 1996), Norwegian (231) (Opdal et al., 1998; Richards et al., 2000), Icelandic (53) (Sajantila et al., 1995; Richards et al ., 1996); North-West Europe - French (71), the British (100) (Piercy et al., 1993), the population of Cornwall (92) (Richards et al., 1996, 2000), the population of Wales (101) (Richards et al., 1996) Irish (West Irlanidiya) (101) (Richards et al., 2000); North-Eastern Europe: Russian (North Caucasus) (25) (Richards et al., 2000), Russians (201) (Malyarchuk et al., 2002), Finns and Karelians (163) (Sajantila et al., 1995; Richards et al., 1996, 2000), Estonian (149) (Sajantila et al., 1995, 1996; Richards et al., 2000), Volga Finns (34) (Sajantila et al., 1995).

**Number of samples analyzed for West Siberian Bronze Age cultural groups (all haplogroups):**

| **Cultural group** | **Number of Samples** |
| --- | --- |
| Ust-Tartas culture | 25 |
| Odinovo culture | 15 |
| Krotovo culture | 6 |
| Late Krotovo culture | 20 |
| Andronovo (Fedorovo) culture | 20 |
| Pakhomovo culture | 10 |
| Total (all cultural groups) | 96 |

**Given the low number of each series, we believe that the calculation of the frequency of haplogroup A10 for each of ancient populations alone will not reflect a reliable picture. In our opinion, a more accurate may be assessment of the A10 frequency in the total series from Western Siberian Bronze Age populations (N = 96), which is 0.104. Taking into account that the pairs of individuals with identical A10 haplotypes from Ust-Tartass and Odinovo groups with a high probability may be close relatives, we exclude one sample of each pair from the sample. As a result, we obtain the sum frequency of not less than 0.0851.**

This frequency is significantly higher than frequency of haplogroup A10 in any of the modern populations. For comparative analysis, we calculated the average frequency of haplogroup A10 in modern indigenous populations of the two main regions of its distribution - Western Siberia and the Volga-Ural region.
To do this, we used the following total sample (see the list above for the references):
Western Siberia - 957 individuals (Khanty - 210 + 106, Komi - 78, Mansi - 98 + 63, + 39 107 Nganasans, chum - 38 Siberian Tatars - 218). The number of carriers of haplogroup A10 - 8 individuals. The average frequency of haplogroup - 0.008.

Volga-Ural region - 1176 individuals (the Komi-Permian - 74, Komi peoples - 62, Udmurtia - 101 Mordvinians - 102 Mari - 136, Bashkirs - 221, Tatars - 228 + 197, Chuvash - 55). The number of carriers of haplogroup A10 - 15 individuals. The average frequency of ~ 0.013.
In other regions of Eurasia frequency of haplogroup A10 even lower.
The frequency of haplogroup A10 significantly exceeds the average rate in modern populations of Western Siberia and the Volga-Ural region (p <0.001 using Student criterion).
