# Supplementary material for: MtDNA Haplogroup A10 Lineages in Bronze Age Samples Suggest That Ancient Autochthonous Human Groups Contributed to the Specificity of the Indigenous West Siberian Population
Source: PLoS One. 2015 May 7;10(5):e0127182. doi: 10.1371/journal.pone.0127182 (PMC4423966; doi:10.1371/journal.pone.0127182)
Supplement: S2 File — (DOC) [file pone.0127182.s005.doc]

**Ust-Tartas culture.**

The Ust-Tartas archaeological culture was discovered in 2001 by Molodin V.I..

**Area of the Culture:**

Forest-steppe belt between the rivers Ob and Irtysh.

**General dating of the culture:**

The end of 5^th^ = the beginning of III millennium BC (according to a series of radiocarbon dates obtained for several burial grounds)*.

**Archaeological sites belonging to the Ust-Tartas culture:**

Basic type of Ust-Tartas culture archaeological sites – burial grounds. Only one settlement was discovered to the present moment. This settlement remains poorly studied. Two largest burial grounds – Sopka-2/3, Sopka-2/3a, which contained most of the presently known Ust-Tartas culture burials (more than 70 burials, more than 100 individuals) have been fully investigated to date. On the other cemeteries (Grishkina Zaimka, Kryuchnoe-6 Krohalevka-13, Tartas-1, Preobrazhenka-6) only single burials were investigated.

**Funeral practice:**

Mainly graves were ground without any gravestones or burial mounds. Single burials (with 1 buried individual) are rare, but a lot of collective graves (the number of buried varies from 2 to 15 individuals). Secondary burials (when human remains in some stage of decompositions were buried) are often presented in the collective burials. Burials arranged in rows oriented on north-south line. Skeletons lie on back, with head on the north-east.

**Archaeological inventory:**

The overall inventory exhibits archaic features. Many types of equipment similar to Neolithic (or even Mesolithic) ones.

Ceramics: Ceramics in burials is extremely rare. Due to the small amouns of the ceramic material, its full characterization is difficult to date.

Stone tools: Stone tools are various: arrowheads of various shapes and sizes, knife-lik plates and knives, axes, adzes, etc.

Bone tools: mainly bone arrowheads and knives (daggers).

Adornments: pendants, animal teeth, stones and shells of mollusks, sewn onto clothing. Ust-Tartas culture adornment are close to Neolithic ones.

Bronze items: Bronze items are very rare in Ust-Tartas archaeological sites, found only two times (in Sopka-2/3a and Tartas-1 cemeteries), in the form of thin bronze platelets. This is only the initial stage of the metal usage in the forest-steppe region of West Siberia.

**The economic type:**

Population of Ust-Tartas culture was typical hunter-gatherers.

**Anthropological type:** Ust-Tartas population demonstrates a combination of craniometric features typical for West Eurasian and East Eurasian populations and belongs to Ural (West Siberian) anthropological type. Ust-Tartas population belongs to the so-called “northern Eurasian anthropological formation”.

**Supposed origin of the Ust-Tartas population:**

Archaeological and anthropological data clearly evidenced an authochthonous origin of the Ust-Tartas population from the preceding Neolithic populations of the Baraba region.

**The paleoanthropological materials from Ust-Tartas population, analyzed in this study:**

For this study bone samples from two individuals from the collective burial N 655 of Sopka-2/3a Ust-Tartas culture burial ground (one of the two largest cemeteries of this culture) were obtained. This burial contained remains of 14 individuals. Skeleton A (male, 40-50 years of age) is central. The remaining skeletons (or parts thereof) were placed around the skeleton of A. In this paper we studied samples from skeletons B (female, 40-45 years, Ut38) and D4 (female, 30-35 years, Ut5).

A series of radiocarbon dates suggests that burial Sopka-2/3, 3a date to the middle of 4 th century BC) [Molodin et al., 2012 and unpublished results].

C14 dates for the samples:

The date was obtained for D2 skeleton from the grave 655:

4695±75, SOAN-8275, 3650BC (95.4%) 3340BC.

**Odinovo culture.**

The Odinovo type of archaeological sites was firstly highlited by M.F. Kosarev in 1976. The Odinovo archaeological culture was discovered by V.I. Molodin in 2008.

**Area of the Culture:**

Forest-steppe zone of Western Siberia, from the basins of the rivers Ishim and Tobol in the west to the Baraba forest-steppe (between rivers Ob and Irtysh) in the east. Rare Odinovo sites located in the southern taiga zone of Western Siberia.

**General dating of the culture:**

3^rd^ millennium BC (according to a series of radiocarbon dates [Molodin et al., 2012 and unpublished results])*.

**Archaeological sites belonging to the Odinovo culture:**

Mainly Odinovo culture settlements discovered in the western part of the overall area of the Odinovo culture. In the eastern part of the area (the Baraba forest steppe) both settlement and large burial grounds (Sopka-2/4a, Preobrajenka-6, Turunovka-1, Abramovo-10, Tartas-1) belonging to the Odinovo culture were investigated. Thus, almost all Odinovo paleoanthropological material available for DNA analysis was obtained from baraba forest-steppe. In total, several dozen of dwellings and more than a hundred burials belonging to the Odinovo culture investigated to date.

**Funeral practice:**

Mainly graves were ground without any gravestones or burial mounds. Most graves are single (with only 1 individual buried), rarely paired and collective graves. Graves are arranged in rows. Skeletons lie on back, with head to the north-east. The head and upper part of body were elevated. Sometimes legs bent at the knees. Vessels were rarely placed in the burial. Fairly simple bone, stone, or metal inventory put in the grave.

**Archaeological inventory:**

In general, Odinovo culture inventory is not as archaic as in prior period. Metal is more widely used for the manufacture of different types of inventory.

Ceramics: Ceramic vessels are rare in the graves, but much more ceramics found in the settlements. Thus, ceramic complex is well characterized. Odinovo ceramics represented by the flat-bottomed jars or slightly profiled vessels ornamented over the entire surface, including the bottom. Often there are textile prints on the internal surfaces.

Stone tools: Stone used for manufacturing arrowheads, clubs, scrapers, abrasives, decorations in the form of zoomorphic figures, etc. Odinovo culture demonstrate a high level of stone processing.

Bone tools: weapons, implements, adornments, symbolic objects of cult.

Bronze items: Odinovo bronze tools are significantly more common and varied than in earlier cultural groups. It represented by Seima-Turbino types of bronze tools (forked spear and other tools), double-edged knives and awls, earrings and pendants. In addition to bronze tools there are rare gold and silver jewelry (earrings). Evidence of the existence of bronze production – molds, crucibles, etc. have been found in the Odinovo settlements. Thus, Odinivi population already has a well-developed production and processing of bronze.

**The economic type:**

Hunting and fishing played the key role in the economy of Odinovo population. Additionally, dirung this period there was a transition to producing economy (appearance of animal husbandry). The latter is evidenced by the findings of cattle and horse bones in Odinovo archaeological sites.

**Anthropological type:** The Odinovo culture population show anthropological similarities with previous populations of Baraba region (Neolithic and Ust-Tartas groups) and belongs to belongs to Ural (West Siberian) anthropological type.

**Supposed origin of the Odinovo population:**

The physical anthropology clearly indicated that Odinovo populations originated on tha basis of the autochthonous anthropological substrate presented in the preceding groups of Baraba forest-steppe region since the Neolithic period [Chikisheva, 2012].

**The paleoanthropological materials from Odinovo population, analyzed in this study:**

For this study bone samples from three Odinovo individuals were obtained. Krz1 – from the burial N 210 of Sopka-2/4a cemetery. This grave has two tiers: two men of 40-50 years of age were buried one above the other. The infant bones were also found in the lower tier. The sample (teeth) were taken from the skeleton of man from lower tier.

Two other samples were taken from individual graves N 10 and 48 of adult humans from Preobrajenka-6 burial ground.

C14 dates for the samples*:

Od7: 3800±100, SOAN-8700, 2550BC (95.4%) 1950BC.

Krz1: 3935±50, SOAN-8687, 2580BC (95.4%) 2280BC.

**Krotovo culture.**

The Krotovo type of archaeological sites was firstly highlighted by V.F. Gening. The Krotovo archaeological culture was discovered by V.I. Molodin in 1975 [Molodin, 1975].

**Area of the Krotovo culture:**

The forest-steppe zone between the rivers Ob and Irtysh (Baraba forest-steppe) and some adjacent territories.

**General dating of the culture:**

Krotovo culture divided into two chronological stages: early stage – Krotovo culture, and late stage – Late Krotovo culture. The early stage of Krotovo culture dated by the 3^rd^ millennium BC (mainly the first half of the 3^rd^ millennium) (according to a series of radiocarbon dates [Molodin et al., 2012 and unpublished data])*.

**Archaeological sites belonging to the Krotovo culture:**

Many settlements and burial grounds, belonging to the Krotovo culture, are well studied to date. Excavations of several settlements (Chernoozerie-IV, Vengerovo-2-3 Preobrazhenka, Krotovo-7/8) allowed to reconstruct Krotovo dwellings. Dwellings represented by semi-dugouts consisting of one or two rooms. Hearth was located in the center of dwelling.

Several Krotovo cemeteries were investigated to date, ranging from small (Ordynskoe-1B) to large burial grounds, containing hundreds of burials (the biggest cemetery - Sopka-2/4B).

**Funeral practice:**

Krotovo graves were ground without any gravestones or burial mounds. Most graves are single (with only 1 individual buried). Graves are arranged in rows. Skeletons lie on back, with head to the north-east (traditionally for the preceded Baraba bronze Age populations). Burial inventory represented by bone, stone and bronze tools and adornments, silver and gold adornments. Ceramic vessels were rare in Krotovo burials.

**Archaeological inventory:**

Ceramics: Ceramic vessels are rare in Krotovo burials. But Krotovo funeral ceramics is more variable, than ceramics from Krotovo settlements. Krotovo ceramic complex is specific and well characterized. Krotovo ceramics is mainly represented by large vessels, demonstrating the specificity of the ornament. Apart from ceramic vessels the ceramic tools for bronze production and processing (molds, crucibles, etc.) were manufactured and used.

Stone tools: Stone tools represented by arrowheads, scrappers, knives and molds.

Bone tools: arrowheads, tools, symbolic objects, armor, etc.

Bronze items: Bronze items have been used widely. Krotovo culture bronze items included weapons, specisic two edged daggers, knives, spears and other tools of Seima-Turbino type, jewelry (including silver and gold ones) such as represented by large temporal rings and earrings. The Krotovo population had a well developed bronze casting production, as evidenced by tools for bronze casting, found by archaeologists.

**The economic type:**

Hunting and fishing played the key role in the economy of the Krotovo population. Animal husbandry was well developed, but plays a minor role.

**Anthropological type:** Anthropological type of the Krotovo culture population was described on materials from the burial ground Sopka-2/4B. The samples from one skeleton from the same burial ground was used in this study. The anthropological type of the Krotovo population was extremely close to the anthropological type of Ust-Tartas and Odinovo populations.

**Supposed origin of the Krotovo population:**

Autochthonous origin of Krotovo population from the preceding human groups of Baraba unambiguously proved by archaeological and anthropological data [Molodin, 1985; Chikisheva, 2013].

**The paleoanthropological materials from Krotovo population, analyzed in this study:**

A sample from the Sopka-2/4B burial ground was analyzed in this study (this burial ground is the most representative for Krotovo population). The sample was obtained from the burial N 177, containing the remains of an adult individual (presumably male).

C14 date for the sample:

4170±80, SOAN-7723, 2920BC (93.2%) 2560BC*.

**Late Krotovo culture.**

The Late stage of the Krotovo culture was described by V.I. Molodin and later was named “Chernoozerskaya culture” [Molodin, 2014]. This cultural group is the final stage of Krotovo culture development, during which the autochthonous Krotovo population intensively interacts with Andronovo (Fedorovo) groups, migrated into the West Siberian forest-steppes from the beginning of II millennium BC.

**Area of the Late Krotovo culture:**

The forest-steppe zone between the rivers Ob and Irtysh (Baraba forest-steppe) (similar to the area of the Krotovo culture).

**General dating of the Late Krotovo culture:**

The Late Krotovo culture dates from the end of the 3^rd^ to the 1^st^ third of the 2^nd^ millennium BC (according to a series of radiocarbon dates [Molodin et al., 2012 and unpublished data])*.

**Archaeological sites belonging to the Late Krotovo culture:**

Several large burial grounds (Sopka-2/5, Chernoozerie-1, Tartas-1) and a settlement (settlement Chernoozerskoe) have been excavated to date. The burial grounds contain a large number of burials and belong to both the early stage of Late Krotovo culture formation (the dominance of the autochthonous features in the material culture, Sopka-2/5 burial ground) and to the phase of active interactions between indigenous and migrated groups (dominance of syncretic features in the material culture, Tartas-1 burial ground).

**Funeral practice:**

Late Krotovo graves were ground, without any gravestones or burial mounds. Graves are arranged in rows. Skeletons lie on back, with head on north-east (traditionally for the preceded Baraba Bronze Age populations). In some syncretic burials skeleton is criched on his side, with head to the east or southeast. Burial inventory represented by bone and bronze artefacts. Ceramic vessels were more common than in the previous periods.

**Archaeological inventory:**

Ceramics: Ceramic vessels were more common in the Late Krotovo burials than in the previous periods of the Bronze Age. A characteristic feature of the late Krotovo ceramic complex was syncretism – the combination of traits that are characteristic of earlier Krotovo and Andronovo (Fedorovo) ceramics.

Stone tools: are less common than in previous periods.

Bone tools: are less common than in previous periods (bone awls, arrowheads, pendants).

Bronze items: Bronze items have been used widely. During this period there was a transition there was a transition from the Seima-Turbino to the Timber-Grave – Andronovo traditions in bronze items production. Late Krotovo culture bronze items included two cutting edges daggers, bracelets, rings and earrings decorated with spiral terminals, asa well as the knife-like pendants.

**The economic type:**

The role of animal husbandry was significantly becomes much more important than in previous Bronze Age periods. At the same time there was a slight decrease of hunting and fishing role.

**Anthropological type:** Anthropological type of the Late Krotovo culture population was described on materials from the burial grounds Sopka-2/5 and Tartas-1. Anthropological type of the Late Krotovo population shows significant differences compared with previous populations of the Bronze Age. These changes were caused by increased specific traits that are characteristic of West Eurasian populations, and some other components. The new genetic components come into the region directly with migrated Andronovo populations and with indigenous populations of neighboring regions of Western Siberia that displaced northward by Andronovo migration wave.

**Supposed origin of the Late Krotovo population:**

Archaeological and anthropological data clarly indicate a syncretic origin of the Late Krotovo population as a result of the interaction of indigenous Baraba populations (Krotovo) and migrant Andronovo (Fedorovo) population.

**The paleoanthropological materials from Krotovo population, analyzed in this study:**

The sample from a Late Krotovo individual for this study was obtained from th Tartas-1 burial ground, which was formed during the period of intensive interactions of Lat Krotovo and Andronovo populations. The material was obtained from the grave number 76, which shows archaeological traits characteristic of Late Krotovo culture. The remains of one adult individual were buried in this grave.

C14 dating of this skeletal remains was not carried out yet.

**Andronovo (Fedorovo) culture.**

Andronovo culture was discovered by S.A. Teploukhov in 1927.

**Area of the Andronovo culture:**

The total area of the Andronovo culture covers a huge area of steppe, forest-steppe and southern taiga zones of Eurasia from the Ural Mountains in the West to the Yenisei river in the east. Fedorovo variant of Andronovo culture is prevailed mainly in the eastern part of the total Andronovo culture area – from the Irtysh river to the Yenisei river. Thus, the baraba forest steppe located in the north-eastern of the total area of Andronovo culture.

**General dating of the Andronovo (Fedorovo) culture:**

The existence of Andronovo (Fedorovo) culture in the Western Siberia dates from the first half of the 2^nd^ millennium BC (according to a series of radiocarbon dates [Molodin et al., 2012 and unpublished data])*.

**Archaeological sites belonging to the Andronovo (Fedorovo) culture:**

Only burial grounds were investigated in the Western Siberia (a few settlement were studied only in the eats of Ural region (Zauralye)). A large number of burial grounds? Both small and very large, has been studied to date. Burials reflecting different stages of the interaction of migrated Andronovo groups with different autochthonous populations (from “pure” Andronovo to syncretic complexes) are presented in the investigated ceneteries.

**Funeral practice:**

Burials usually with earthen mounds (barrows), but sometimes without it (ground). In Funeral practice of Andronovo culture included two main burial rites – cremation and inhumation. In the latter case, the buried individuals are crouched on the left side, head to the southwest. Burial items represented mainly by vessels (1, 2 or more), which were placed in each grave in the area of human head. In contrast to previous periods, other equipment (weapons, tools, jewelry) were rarely put in the grave.

**Archaeological inventory:**

Ceramics: Ceramic vessels are necessarily present in all burials (1.2 or more vessels). Ceramic complex is characterized by a distinct specificity ornamentation that distinguishes it from previous ceramic previous West Siberian cultural groups.

Stone tools are rarely presented in Andronovo burials.

Bone tools are rarely presented in Andronovo burials.

Bronze items: Bronze items are rarely occur in Andronovo burials. Bronze inventory represented by daggers of Timber Grave-Andronovo (Srubno-Andronovo) type, jewelry (bracelets with spiral terminals, earrings with bells).

**The economic type:**

The animal husbandry dominates in the Andronovo culture economy. Hunting and fishing play a minor role.

**Anthropological type:** Anthropological type of Andronovo population differs strongly from the previous West Siberian human groups. It is slightly varies in different regions of Western Siberia. The West Eurasian (proto-Caucasian) anthropological types form its basis. East Eurasian craniometrical traits were represented also to varying degrees in local Andronovo groups. Variability of anthropological characteristics is explained by different sources of migrated Andronovo populations and various nature and degree of interaction with the autochthonous West Siberian human groups. The greatest degree of genetic interaction with indigenous populations observed for Andronovo groups from the Baraba forest-steppe.

**Supposed origin of the Andronovo (Fedorovo) population:**

Archaeological and anthropological data clearly show migrant origin of Andronovo populations in Western Siberia. At the same time, Andronovo populations in most local areas of Western Siberia were showing signs of assimilation with aboriginal human groups, i.e. it were already mixed.

**The paleoanthropological materials from Andronovo (Fedorovo) population, analyzed in this study:**

One bone sample from the Tartas-1 Andronovo (Fedorovo) burial ground was analyzed in this study. This cemetery was formed in the period of active interaction of indigenous Krotovo and migrated Andronovo populations. Andronovo materials from the Tartas-1 burial ground are among the most representative in the Western Siberia and reflect a high degree of interaction with the autochthonous Late Krotovo population. The bone sample for the study was obtained from the burial № 189. One adult individual, presumably female, was buried in this grave.

C14 date for the sample: SOAN-7117, 3310+_30; 1680(95,4%)1510 BC*.

**Pakhomovo culture.**

Pakhomovo culture was discovered by O.N. Korochkova in 1987 based on archaeological materials from a region between Tobol and Irtysh rivers [Korochkova, 1987]. Archaeologically close materials from the Baraba forest-steppe were attributed to the eastern variant of the Pakhomovo culture in 2012 [Molodin, 2012].

**Area of the Pakhomovo culture:**

The area of the Pakhomovo culture covers a forest-steppe zone from the Tobol raver in the West to the central part of the Baraba forest-steppe in the east.

**General dating of the Pakhomovo culture:**

The existence of the Pakhomovo culture in the Western Siberia dates from 14^th^ to 10^th^ centuries BC (or even to 8^th^ centuries BC) based on archaeological inventory dating.

**Archaeological sites belonging to the Pakhomovo culture:**

Pakhomovo culture sites are unevenly distributed throughout total area of the culture. In the western part of the area mainly settlements were discovered (Pahomovskaya Pristan I, Inberen IV, Novo-Shadrino VII, Uk III) and a small number of burials. In the eastern part of the area (Baraba forest-steppe) there are no known Pakhomovo settlements. However, most of the Pakhomovo burials were studied to date are located in the Baraba region.

**Funeral practice:**

Burials were made under the mound. Both single and collective graves investigated in Pakhomovo culture cemeteries. There were many secondary burials. Position of the buried body varies – sometimes on the back, sometimes crouched on the side. Ceramic vessels and less often other inventory were placed in the burials.

**Archaeological inventory:**

Ceramics: Ceramic vessels present in many of the burials. Ceramic ornamentation demonstrates clearly visible Andronovo motives, with modifications.

Bone tools are presented by bone arrowheads, end plates on the bow, borers and awls.

Bronze Items: awls, spherical metal pleates with pin or loop, the kelts with bushing [Matveeva, Kostomarov, 2008, p. 425], two-bladed knives and socketed arrowheads, beads. On settlements ceramic crucibles with traces of melting bronze, as well as mold were found.

**The economic type:**

The animal husbandry dominates in the Pakhomovo culture economy. Hunting and fishing play a minor role.

**Anthropological type:** Anthropological type of Pahomovo population is similar to other post-Andronovo human groups of Western Siberia and is close to the anthropological type of Andronovo population.

**Supposed origin of the Pakhomovo population:**

Archaeological and anthropological data clearly indicate the origin of Pahomovsky population as a result of interaction between migrated into the region Andronovo populations and indigenous Bronze Age populations in Western Siberia (as well as other post-Andronovo tribes). Groups of migrants (that are also descendants of local Andronovo groups) from the South (modern Kazakhstan) probably also participate in the formation of the post-Andronovo populations in Western Siberia, including Pakhomovo population.

**The paleoanthropological materials from Pakhomovo population, analyzed in this study:**

Two samples from the Stariy Sad burial ground (the Baraba forest-steppe) were used for this study. The Satariy Sad is the largest of the Pakhomovo culture cemeteries known to date. The samples were obtained from two graves: grave 1 of kurgan 6, and grave 1 of kurgan 49. Both the analyzed individuals – adults. C^14^ dating of these materials has not yet been evaluated.

*******Currently, our colleague-archaeologists are working hard on radiocarbon dating of the Bronze Age material from the territory of Western Siberia. In the manuscript we have presented the C14 data on age of ancient populations we studied provided to us by our collegues (Tables 1, 2, Supplementary file 5). Many of the dates on which these estimates are based, have not been published yet, and can not be given by us in this manuscript. The data that have been published, including the numbers of dates, links to the lab, etc. presented in related publications [Molodin et al., 2012]. All C14 dates were calibrated by programm OxCal_3.10 [Reimer et al., 2004].. Also, when calculating the correction for interior reservoir effect. Thus, the data of radiocarbon dating provided to us, meet generally accepted criteria in this area.

Molodin V.I., Marchenko Z.V., Kuzmin Y.V., Grishin A.E., Van Strydonck M., Orlova L.A. 14C Chronology of Burial Grounds of the Andronovo Period (Middle Bronze Age) in Baraba Forest Steppe, Western Siberia // Radiocarbon. – 2012. – Vol. 54, № 3-4. – P. 737-747.

Reimer P.J., Baillie M.G.L., Bard E., Bayliss A., Beck J.W., Bertrand C.J.H., Blackwell P.G., Buck C.E., Burr G.S., Cutler K.B., Damon P.E., Edwards R.L., Fairbanks R.G., Friedrich M., Guilderson T.P., Hogg A.G., Hughen K.A., Kromer B., McCormac G., Manning S., Bronk Ramsey C., Reimer R.W., Remmele S., Southon J.R., Stuiver M., Talamo S., Taylor F.W., van der Plicht J., Weyhenmeyer C.E. IntCal04 terrestrial radiocarbon age calibration, 0–26 cal kyr BP // Radiocarbon. – 2004. – Vol. 46, № 3. – P. 1029–1058.
